# Endometriosis MR mimickers: T2-hypointense lesions

**DOI:** 10.1186/s13244-023-01588-2

**Published:** 2024-01-25

**Authors:** Edouard Ruaux, Wendaline M. VanBuren, Stéphanie Nougaret, Marie Gavrel, Mathilde Charlot, Flavia Grangeon, Pierre-Adrien Bolze, Isabelle Thomassin-Naggara, Pascal Rousset

**Affiliations:** 1 Department of Radiology, Hospices Civils de Lyon, Lyon Sud University Hospital, Lyon 1 Claude Bernard University, EMR 3738, Pierre Bénite, France; 2 Department of Radiology, Mayo Clinic, Rochester, MN55905 USA; 3 Department of Radiology, Montpellier Cancer Institute, U1194, Montpellier University, 34295 Montpellier, France; 4 Department of Gynecology and Obstetrics, Hospices Civils de Lyon, Lyon Sud University Hospital, Lyon 1 Claude Bernard University, EMR 3738, 69495 Pierre Bénite, France; 5 Department of Radiology, Service Imageries Radiologiques Et Interventionnelles Spécialisées, Hôpital Tenon, Assistance Publique Hôpitaux de Paris, Sorbonne Université, 75020 Paris, France

**Keywords:** Endometriosis, Deep infiltrating endometriosis, Pelvic inflammatory disease, Genital diseases, Magnetic resonance imaging

## Abstract

Endometriosis is a common crippling disease in women of reproductive age. Magnetic resonance imaging (MRI) is considered the cornerstone radiological technique for both the diagnosis and management of endometriosis. While its sensitivity, especially in deep infiltrating endometriosis, is superior to that of ultrasonography, many sources of false-positive results exist, leading to a lack of specificity. Hypointense lesions or pseudo-lesions on T2-weighted images include anatomical variants, fibrous connective tissues, benign and malignant tumors, feces, surgical materials, and post treatment scars which may mimic deep pelvic infiltrating endometriosis. False positives can have a major impact on patient management, from diagnosis to medical or surgical treatment. This educational review aims to help the radiologist acknowledge MRI criteria, pitfalls, and the differential diagnosis of deep pelvic infiltrating endometriosis to reduce false-positive results.

**Critical relevance statement**

MRI in deep infiltrating endometriosis has a 23% false-positive rate, leading to misdiagnosis. T2-hypointense lesions primarily result from anatomical variations, fibrous connective tissue, benign and malignant tumors, feces, surgical material, and post-treatment scars.

**Key points**

• MRI in DIE has a 23% false-positive rate, leading to potential misdiagnosis.

• Anatomical variations, fibrous connective tissues, neoplasms, and surgical alterations are the main sources of T2-hypointense mimickers.

• Multisequence interpretation, morphologic assessment, and precise anatomic localization are crucial to prevent overdiagnosis.

• Gadolinium injection is beneficial for assessing endometriosis differential diagnosis only in specific conditions.

**Graphical Abstract:**

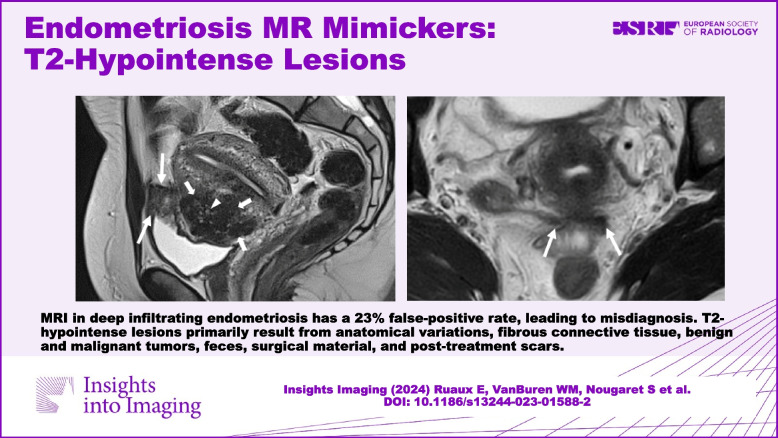

**Supplementary Information:**

The online version contains supplementary material available at 10.1186/s13244-023-01588-2.

## Background

Endometriosis is a common disease of reproductive-age women related to endometrial-like tissue outside the uterine cavity with an estimated prevalence between 5 and 10% [1]. Three clinical phenotypes of endometriosis may coexist: ovarian cysts known as endometriomas, superficial peritoneal implants, and deep infiltrating endometriosis (DIE) [2]. DIE is defined as an extension of endometrial tissue-like below the peritoneal surface, with the ability to invade adjacent structures, associated with fibrosis and disruption of normal anatomy [2]. DIE can affect almost any organ or structure, although most lesions are found in the pelvic region, especially the posterior compartment [3]. Patients usually present with chronic pelvic pain, severe dysmenorrhea, dyspareunia, dyschezia, dysuria, and infertility [4], depending on the anatomic location and degree of infiltration, all of which can strongly affect quality of life [5]. This disease represents a public health issue, with a major impact on quality of life, highlighting the importance of an accurate and precise early diagnosis [6]. While transvaginal ultrasonography can be a first-line imaging modality [7], magnetic resonance imaging (MRI) is the cornerstone imaging technique in the evaluation of endometriosis, especially for DIE, with an overall sensitivity of 94% [3]. Dedicated DIE ultrasound protocols by experts have been shown to have a similar sensitivity for certain disease locations, predominantly the ovaries, uterosacral ligaments (USLs), and bowel, but are not widely available [8]. MRI allows exhaustive mapping of DIE lesions, determining the extent of disease and organ involvement, helping gynecologists in the decision-making for a medical or surgical management [9]. However, MRI may lack specificity, leading to a 10% false positive rate overall [10], which increases to 23% in cases of DIE when compared to surgical findings [3]. In the dedicated recommended MRI protocol [11], DIE lesions are well-identified as T2-hypointense solid nodular or fibrotic thickening lesions, with potential associated microcystic or hemorrhagic foci due to the presence of active ectopic glandular tissue [12]. However, other pelvic conditions including in particular anatomic variations or infectious diseases, can exhibit T2-hypointense findings similar to DIE, which may lead to misdiagnosis. Moreover, the challenge may be heightened as the MRI pattern of DIE may also depend on the 'age' of the lesion as well as the degree of fibrosis. While medical history, symptoms, clinical examination, and the presence or absence of other pelvic endometriotic lesions on MRI can assist in diagnosing endometriosis, it is important to acknowledge that several challenges and potential pitfalls still exist in achieving an accurate diagnosis. Misdiagnosis of endometriosis at the initial presentation can result in improper medical or surgical treatments and have significant psychological effects on the patient. Additionally, mistaking other T2-hypointense findings for endometriosis in confirmed cases can overestimate the disease's extent and potentially lead to inappropriate decision-making and interventions.

This review aims to offer valuable tips for distinguishing DIE from other pelvic conditions that exhibit T2-hypointense tissue-like findings. The approach involves interpreting the lesion signal across multiple sequences, conducting morphologic analysis, and ensuring precise anatomic localization.

## T2-hypointense lesions or condition-like lesions mimicking DIE

Various conditions with T2-hypointense tissue-like formations can mimic DIE. These conditions typically exhibit features such as hypointense thickening, nodules, or infiltrating masses that invade pelvic structures or organs, leading to morphological changes and the loss of the normal signal, particularly in the muscularis layer of the affected organ (Table 1).

**Table 1:** T2-hypointense mimickers: MRI key features

Nature of the T2-hypointensity	Structure involved and/or type of condition	MRI key features
**Anatomical variation and pitfalls**		
	**Uterosacral ligaments**	Mostly pseudonodular and/or between 3 to 5 mm thickness, without hemorrhagic foci on T1 FS-WI, use of multiple planes or multiplanar reconstruction on 3D T2-WI Previous history of pelvic surgery and/or upper genital infection
	**Round ligaments**	Mostly pseudonodular and/or < 1 cm, use of multiple planes or multiplanar reconstruction on 3D T2-WI, regular aspect without hyperintense implant on T1 FS-WI Anatomical variation: association with veinous structures (varicosities)
	**Urachus**	Mostly seen on moderately filled bladder, triangular aspect on sagittal T2-WI plane Respect of the muscular layer of the bladder, no hemorrhagic foci on T1 FS-WI
	**Uterine contraction**	Myometrial pseudonodular low signal intensity on T2-WI at the level of the serosa Partial or complete resolution on different planes or repeated acquisition after a suitable interval
**Fibrous tissue**		
	**Vesicouterine pouch**	
	*Cesarean scar*	Linear scar defect of variable thickness, sometimes pseudonodular, up to the pelvic wall Intra- or extra-mural isthmocele + / − retained blood content Absence of external adenomyosis, bladder wall invasion or hemorrhagic foci on T1 FS-WI
	**Pelvic wall**	
	*Round ligaments ligamentoplasty*	Uterus anteversion, shortened round ligaments with a medial course and pseudonodular thickening up to their pelvic wall insertion, no hemorrhagic foci on T1 FS-WI
	Desmoid tumor*	Intermediate signal intensity areas on T2-WI, with high signal intensity on DWI, and intense contrast-enhancement + / − fascial tail sign (inconsistent) Varying size (may be large), ill or well-defined Absence of microcystic structures on T2-WI or hemorrhagic foci on T1 FS-WI
	**Infectious conditions**	
	Actinomycosis*	Solid component masses in low to intermediate signal intensity on T2-WI Necrosis with moderate to high signal intensity on T1 FS-WI and peripheral enhancement and/or micro-abscess Infiltrating and inflammatory stranding pattern of other pelvic structures/organs
	Alveolar echinococcosis* (extremely rare)	Mostly infiltrating masses, high signal intensity microcystic changes on T2-WI No hemorrhagic foci on T1 FS-WI, calcifications may be seen on CT Co-existence of hepatic disease (multicystic infiltrative masses)
	*Past history of pelvic infection or peritonitis*	USLs with mostly pseudonodular aspect < 5 mm, using other planes or multiplanar reconstruction on 3D T2-WI, without hemorrhagic foci on T1 FS-WI
**Benign tumors**		
	**Pelvic organs**	
	Leiomyomas*	Rounded or oval well-defined masses Low (or intermediate) signal intensity on T2-WI without hemorrhagic foci on T1 FS-WI Exophytic growth may be seen without any retraction
**Malignant tumors**		
	**Rectosigmoid**	
	Colorectal carcinoma*	Intrinsic endoluminal lesion with polypoid, semi-circumferential or circumferential morphological aspect, mesorectum infiltration, and tumor deposits High signal intensity with high-b values on DWI (and low ADC)
**Surgical material**	**Ureteral meatus and parameters**	
	*Vesicoureteral reflux treatment*	Geometrical shaped structures at the ureterovesical junction or a little behind Commonly bilateral and symmetrical Collagen materials in low signal intensity on T2-WI ± surrounding granulomas Macroplastiques in iso or hyposignal on T1 FS-WI Hyperdense structures may be seen on CT
	**Urethra**	
	*Periurethral incontinence treatment*	Bulking agent around or within the wall of the urethra in low signal intensity on T2-WI Bulking agent in iso or hyposignal (or not seen) on T1 FS-WI Hyperdense structures may be seen on CT (around the urethra, under the bladder)
**Feces**	**Rectosigmoid**	Endoluminal digestive location on other planes or multiplanar reconstruction on 3D T2-WI Feces-like signal on T1 FS-WI, gas with signal void in low signal intensity on T1-WI

^*^Indicates conditions where gadolinium injection can enhance diagnostic accuracy

## Anatomical variations

### Uterosacral ligaments

The uterosacral ligaments (USLs) originate from the torus uterinus, located in the retrocervical area on the posterior surface of the cervix and upper vagina. They extend backward towards the sacrum, marking the upper boundaries of the posterior cul-de-sac, also referred to as the pouch of Douglas. It is a typical location of DIE, reported in a study as the second most frequently involved entity, following the ovaries [13]. MRI diagnostic performance for torus and USLs in endometriosis is excellent [11]. However, MR imaging is highly sensitive with variable specificity (84%) [3]. Specificity may be increased in combination with clinical examination and/or transvaginal sonography [14]. A recent MRI consensus lexicon on deep pelvic infiltrating endometriosis suggests positive MR features of USL involvement [9]. These features including nodular aspect in two different planes, and/or retraction, and/or thickness > 5 mm, and/or hemorrhagic foci aid in precise and confident diagnosis of USLs involvement in DIE. On the contrary, an asymmetrical aspect, and/or linear thickening ranging from 3 to 5 mm in thickness, and/or irregular margins and/or pseudo-nodular appearance (defined as present in only one plane) are considered equivocal and not specific. These findings can be either a variant or attributed to other conditions, making the diagnosis less definitive [9]. Radiologists should be aware of the potentially non-specific nature of T2-hypointense findings in the absence of hemorrhagic implants or nodules.

**Fig. 1:**
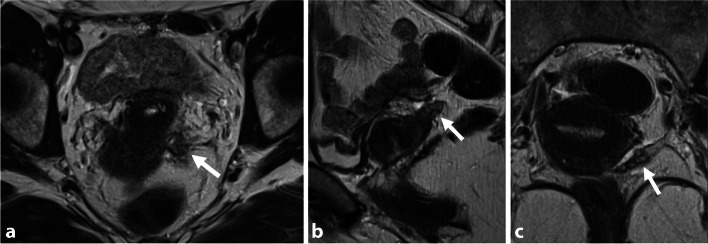
Left utero-sacral ligament (USL) varicosity in a 46-year-old woman with chronic catamenial pelvic pain. **a** Axial, (**b**) sagittal, and (**c**) coronal T2-W MR images show a thickened and pseudonodular left USL (arrows) due to tubular and serpiginous T2-hyperintense veinous structures. No pelvic endometriosis was found at surgery; a pelvic venous congestion syndrome was then suggested

**Fig. 2:**
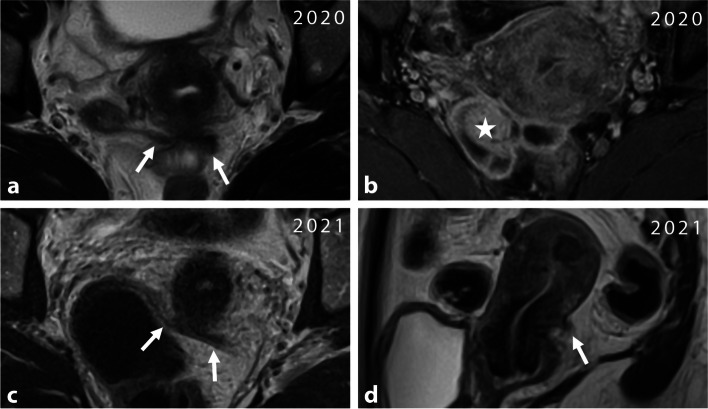
Thickening of the utero-sacral ligaments (USLs) in a 44-year-old woman with history of PID. *Acute episode in 2020:***a** Axial T2-W MR image shows irregular and pseudo-nodular thickening of bilateral USLs (arrows). **b** Axial T1-W fat-suppressed contrast-enhanced MR image shows pyosalpinx (star) with thick-walled fallopian tube and surrounding fat stranding. *One year follow-up in 2021:***c** Axial and (**d**) sagittal T2-W MR images show persistence of pseudonodular thickening of the utero-sacral ligaments (arrows) and the torus, without hemorrhagic foci (not shown)

On one hand, thickened appearance of USLs can be attributed to anatomical variants with asymmetries, varying degrees of fibrous tissue, and regional veins (Fig. 1). It is important to correlate these findings with physical examination and/or ultrasonography. Furthermore, when evaluating the USLs, a past medical history of pelvic inflammatory disease (such as salpingitis or tubo-ovarian abscess) and intestinal diseases (like Crohn's disease or previous peritonitis) should be taken into consideration. These conditions can involve the USLs and lead to post-inflammatory scar thickening (Fig. 2).

### Round ligaments

Round ligaments are intra- and extraperitoneal fibro-muscular structures that extend from the antero-central and antero-lateral pelvic compartments. On MRI, round ligaments are visualized as regular structures with low signal intensity on both T1-weighted (T1-W) and T2-weighted (T2-W) sequences, extending from the uterine horns to the inguinal canals to attach to the vestibule. Endometriosis involvement in the round ligaments is more commonly observed in the proximal third of the ligament, adjacent to the uterus. The right round ligament is often more affected than the left due to retrograde implantation of endometrial tissue in the peritoneal cavity. In cases of a large lesion, it is frequently associated with external adenomyosis [15]. More rarely, DIE involves the extra-pelvic segment within the canal of Nuck [16].

**Fig. 3:**
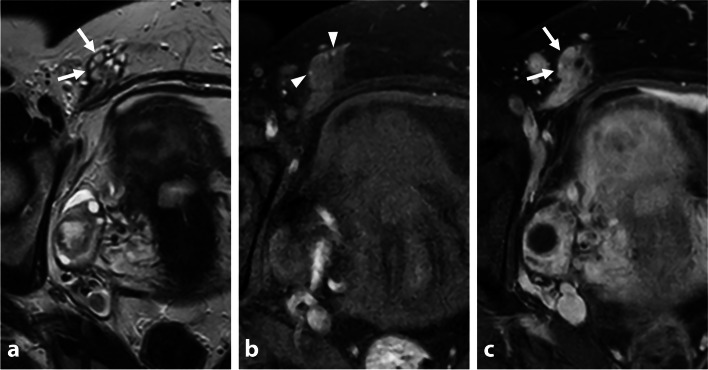
Right round ligament varicosity in the inguinal canal in a 33-year-old woman. **a** Axial T2-W MR images show pseudocystic changes of the right round ligament in its inguinal course (arrows). Thickening appears regular, without any fluid around the ligament. **b** Axial fat-suppressed T1-W MR image reveals a few T1-hyperintense foci (arrowheads) within the right round ligament, due to an “entry slice phenomenon artifact”. **c** Axial T1-weighted fat-suppressed contrast-enhanced MR image shows homogeneous enhancement of tubular veins around the right round ligament (arrows)

The involvement of endometriosis in the round ligaments lacks a consensus definition. However, lesions are commonly observed as nodular (> 1 cm) with irregular margins and varying degrees of microcystic hemorrhagic foci [9]. It is important to note that identifying endometriosis in this location is challenging due to the lack of specificity caused by anatomical variations and the absence of an accurate definition for physiological thickness (Supplemental—Fig. 1). MRI may show tubular or serpiginous structures along thickened round ligaments (and USLs), that may also show T1 bright spots which could potentially be attributed to the “entry slice phenomenon” artifact, a pitfall leading to overdiagnosis (Fig. 3) [17].

### Urachus

**Fig. 4:**
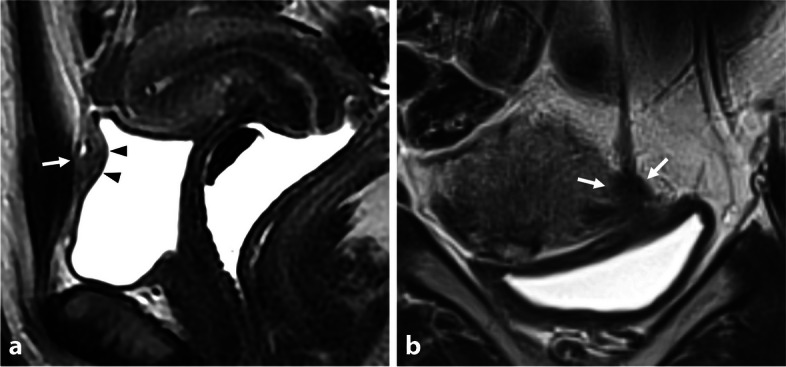
Fibrous remnant of the urachus insertion in two distinct women of reproductive age. **a** Sagittal and (**b**) coronal T2-W MR images show a pseudonodular or triangular T2-hypointense medial structure of the urinary bladder apex at the urachus insertion (arrows). Note the absence of abnormality of the urinary bladder muscular layer (arrowheads), or hemorrhagic foci on T1-WI (no shown)

Imaging findings of urachal remnants typically manifest as T2-hypointense fibrotic tissue due to the presence of dense collagen deposition. However, a large fibrotic remnant can be mistaken for endometriosis of the bladder wall, especially if the bladder is not adequately filled (empty or not full enough) during MR acquisition. Conversely, this aspect is minimized when the bladder is overly distended. The specific anatomical location on the sagittal plane may suggest fibrotic thickening with low T2-W signal intensity. The morphological appearance of the urachus insertion, forming a triangular shape in continuity with the subperitoneal anatomical course, and respect of the bladder muscularis layer intact on T2-W sequences aid in identifying this variant (Fig. 4).

### Uterine contraction

**Fig. 5:**
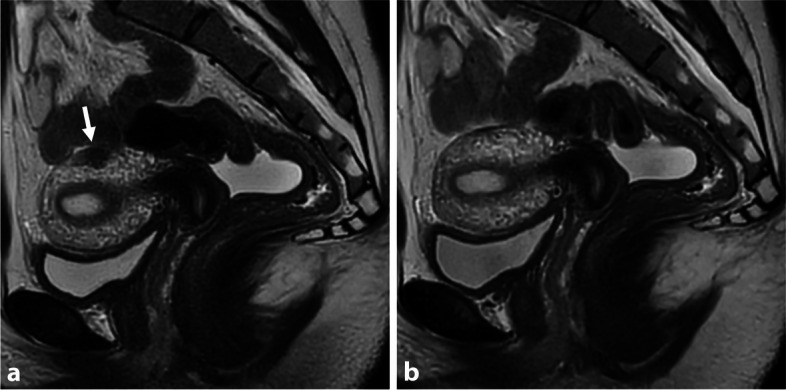
Uterine contraction in a 19-year-old woman with chronic catamenial pelvic pain. **a** Sagittal T2-W MR image shows a T2-hypointense focal thickening (arrow) of the outer myometrium on the back wall of the uterus. **b** Note the absence of abnormality of the myometrium and its complete resolution on additional T2-W MR sequences repeated at the end of the exam

Transient myometrial contraction is a common physiological phenomenon that can mimic pathological conditions such as focal or diffuse adenomyosis [18]. On MRI, it appears as a T2-hypointense region within the outer myometrium, potentially leading to bulging pseudo-thickening of the junctional zone, which can be confused with internal adenomyosis or DIE (Supplemental –Fig. 2). In some cases, it can also be misleading for external adenomyosis, presenting as a pseudonodular T2-hypointense aspect at the level of the serosa (Fig. 5). The key finding to differentiate between these conditions is to compare T2-W images from different planes to assess for partial or complete resolution, as contractions typically improve or resolve between sequences. T2 cine-mode MRI is preferable for evaluation. It is worth noting that while myometrial contraction is transient, it can be sustained for up to 30 ± 45 min, in which case repeating a sequence at that time may be necessary [19].

## Fibrous tissue

Fibrous tissue comprises low-cellularity material in combination with spindle, oval, or round cells resulting in collagen formation. Fibrosis typically demonstrates intermediate signal intensity on T1-WI and very low signal intensity on T2-WI [20].

### Post-surgical scars

#### Cesarean scar

**Fig. 6:**
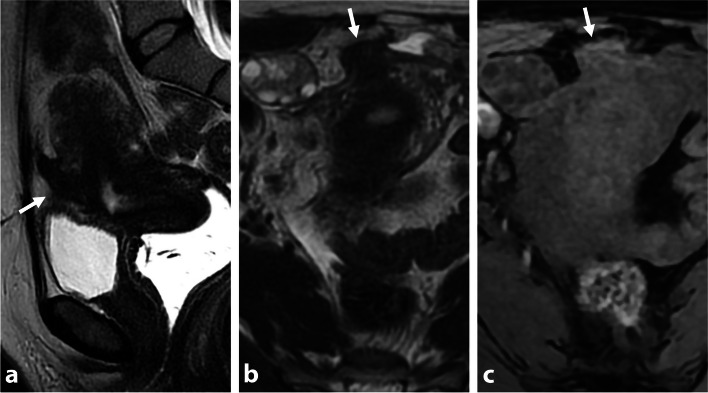
Anterior adhesions in the vesicouterine pouch after C-section in a 40-year-old woman with medical history of pelvic endometriosis. **a** Sagittal and (**b**) axial T2-W MR images show a T2-hypointense fibrous thickening of the vesicouterine pouch, with severe adhesions of the uterine body (arrows). **c** Axial T1-W fat-suppressed MR image shows no hemorrhagic foci in the anterior subperitoneal space (arrow). Endometriosis surgical management has been decided. During surgical procedure, pelvic anterior symphysis was proven with no obvious endometriosis lesion (confirmed with negative biopsies at pathology)

Uterine scar defects can occur in up to 50% of women with infertility and prior cesarean section (C-section) [21]. Surgical interventions like C-section may result in focal adhesions in the vesicouterine pouch, sometimes causing complete obliteration of the anterior peritoneal spaces. Distinguishing between fibrous scar tissue and endometriosis can be difficult in these cases. Post-surgical scars without endometriosis typically appear linear or pseudonodular without external adenomyosis, bladder wall invasion, or hemorrhagic foci (Fig. 6). In some instances, intra- or extra-mural isthmocele in the lower anterior uterine wall with retained blood content may be associated and mistaken for endometriosis [22].

#### Ligamentoplasty

Surgical procedures involving the uterine ligaments, such as ligamentoplasty of the round ligaments and/or uterosacral ligaments, can present challenges in MRI interpretation. After retroversion and hysteropexy surgeries associated with Master Allen syndrome, round ligament ligamentoplasty (which aims to shorten the ligaments and antevert the uterus) can appear as a scar-like, pseudo-nodular changes at their insertion to the pelvic wall, slightly medial to their original course in the inguinal canal [23]. The kinking of the uterosacral ligaments may create a closure of the pouch of Douglas with a pseudo-nodular aspect [24]. These findings are usually isolated, following the anatomical courses of the ligaments, and do not exhibit hemorrhagic foci on T1-WI (Supplemental –Fig. 3).

### Benign tumors

#### Desmoid tumors

**Fig. 7:**
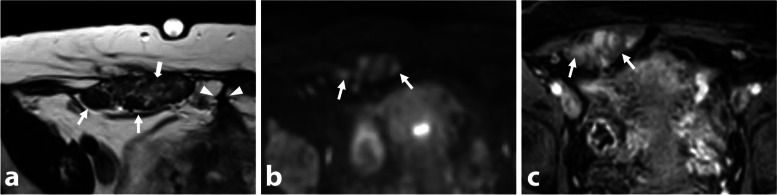
Sporadic pelvic wall desmoid tumor in a 30-year-old woman with history of cesarean section. **a** Axial T2-W MR image shows an infiltrative mass of the right rectus muscle with heterogeneous T2 signal intensity varying from low (thin arrows) to intermediate (thick arrow) signal intensity areas. Anterior focal adhesions due to previous cesarean section are seen (arrowheads). **b** Axial diffusion-weighted MR image shows high signal intensity within the mass consistent with increased cellularity (arrows). **c** Axial T1-W fat-suppressed contrast-enhanced MR image shows heterogeneous enhancement (arrows)

Desmoid fibromatosis is a locally aggressive benign tumor that can occur within the abdominal wall, internally in the abdomen and pelvis (often mesenteric), or extra-abdominal locations [25]. Most desmoid tumors are sporadic and have a predilection for women of reproductive age, with a female-to-male ratio of 3:1 [26]. Sporadic lesions can affect surgical scars and have an unpredictable natural course, which can involve rapid enlargement, spontaneous decrease in size, or resolution. Inheritance plays a role in up to 15% of desmoid fibromatosis tumors, which are associated with familial adenomatous polyposis–related syndromes as Gardner syndrome and Turcot syndrome [27]. Distinguishing a desmoid tumor within the anterior pelvic wall from abdominal wall endometriosis involving C-section scar tissues or laparoscopic port sites can be challenging. Both lesions may exhibit T2-hypointensity with irregular margins and an infiltrating pattern. Desmoid tumors typically display intermediate signal intensity on T2-WI due to increased cellularity, along with high signal intensity on diffusion-weighted imaging (DWI) and intense contrast enhancement. A “fascial tail sign” may be present inconsistently, characterized by thickening and enhancement of the aponeurosis (Fig. 7) [28]. In contrast to intrabdominal DIE lesions, which often exhibit fibrosis and delayed enhancement, wall endometriosis typically shows early and avid enhancement. Besides cyclic pain, key differentiating findings include the absence of hemorrhagic foci or microcystic structures in desmoid tumors. Obtaining tissue samples through US-guided percutaneous biopsies can assist in achieving a definitive histological diagnosis, particularly before or during minimally invasive treatments like percutaneous cryotherapy.

#### Myoma

Leiomyomas, also known as uterine fibroids, can develop in various pelvic structures composed of muscular tissue, including the uterus, vagina, rectum, or urinary bladder (Supplemental –Fig. 4). They usually do not present a diagnostic dilemma, due to their T2-hypointense rounded morphology. However, small-sized leiomyomas with poorly circumscribed margins or an extra-uterine location (especially in cases of prior morcellation) can be confusing, particularly if there are areas of cystic degeneration that may resemble glands. On MRI, leiomyomas typically appear as rounded or oval structures with a homogeneous T2-hypointense signal within the muscularis layer of the uterus, well-defined margins, and the absence of hemorrhagic foci on T1-WI (Supplemental –Fig. 5). The absence of extrinsic infiltration or any associated retraction on T2-W sequences may help in the differential diagnosis.

### Malignant tumors

#### Colorectal carcinomas

Colorectal cancer is the second most common cancer in women [29]. Prevalence of colorectal carcinoma increases with age. However, up to one-third of the patients under 40-year-old have been reported to be linked to hereditary syndromes, such as Lynch syndrome [30]. Nonspecific clinical findings, like rectal bleeding and rectal syndrome, can be misleading during a physical examination. These symptoms alone may not provide a clear indication of the underlying cause, as clinical exam findings are often non-specific and rectal bleeding may not always be present.

**Fig. 8:**
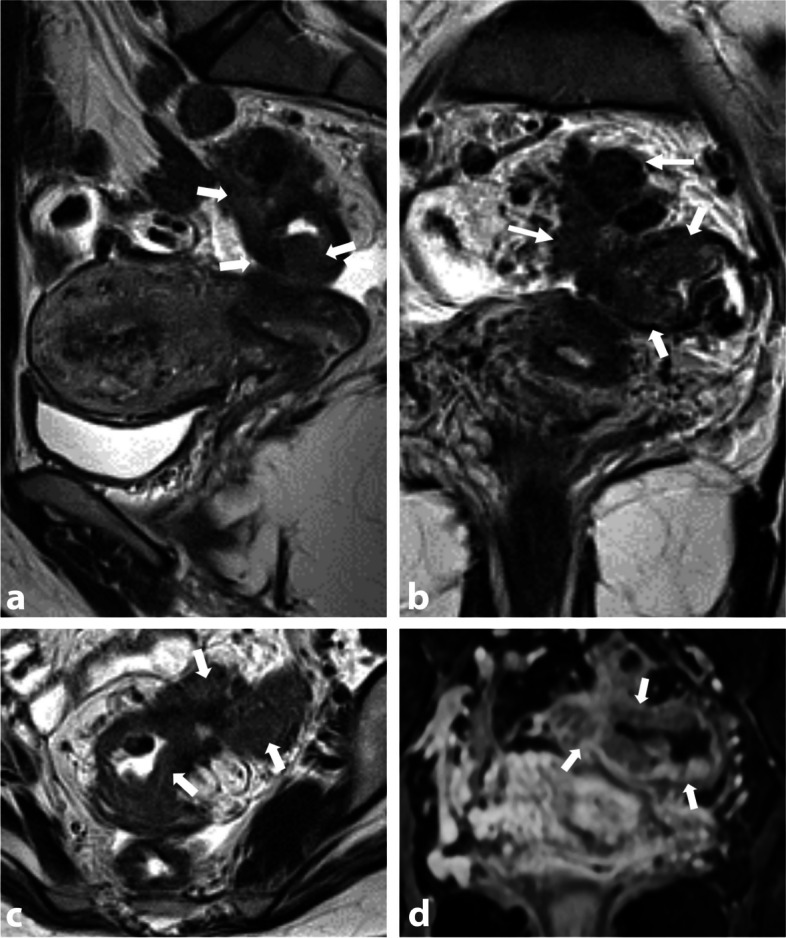
Rectal adenocarcinoma in a 33-year-old patient with chronic pelvic pain and rectal disorder with rectal bleeding, addressed for suspicion of endometriosis. **a** Sagittal, (**b**) coronal, and (**c**) axial T2-W MR images show a T2-hypointense focal wall thickening of the high rectum (thick arrows) with a T2-hypointense tumoral infiltration of the mesorectum (thin arrows). Note the absence of retrocervical deep infiltrating endometriosis. **d** Axial T1-W fat-suppressed contrast-enhanced MR image shows a moderately enhanced tumor (arrows)

Rectosigmoid endometriosis has a distinct morphological pattern different from colorectal cancer. Colorectal cancer typically presents with polypoid, circumferential, and/or semi-circumferential lesions that originate from the mucosa and invade the inner layers. In contrast, rectosigmoid endometriosis rarely exhibits circumferential growth pattern or mucosal invasion [12]. Instead, it begins at the serosa and develops a specific “mushroom cap” sign over time, providing a highly specific indication [31]. Busard et al.proposed a qualitative assessment of high b-value on DWI as a valuable, non-invasive tool to facilitate differentiation between endometriosis infiltrating the bowel and colorectal carcinoma [32]. They both demonstrate low ADC (apparent diffusion coefficient) values. Colorectal carcinoma shows high signal intensity on DWI due to high cellularity (true restricted diffusion), whereas endometriosis displays hypointense signal intensity due to the “T2-blackout effect” of these lesions on DWI (Fig. 8).

### Infectious conditions

#### Actinomycosis

Actinomycosis is a chronic granulomatous disease and bacterial infection caused by *Actinomyces* species. Infections of the female genital tract with *Actinomyces* represent 20% of cases [33] and may be caused by surgery, perforation of the bowel, or foreign bodies, such as intrauterine devices (IUD) [34]. Actinomycosis associated with an IUD typically affects the pelvic area and affected patients often present with chronic pelvic pain and insidious symptoms [35]. Pelvic actinomycosis can extend extensively, reaching a severity comparable to that of a frozen pelvis, which can resemble pelvic malignancy or endometriosis [36]. The intraabdominal extension typically occurs through contiguous spread, as the actinomycosis bacteria produce proteolytic enzymes that enable crossing of normal anatomical barriers. This can result in an infiltrating retractile pattern with firm fibrotic tissue, and in some cases, the formation of abscesses and/or fistulas.

**Fig. 9:**
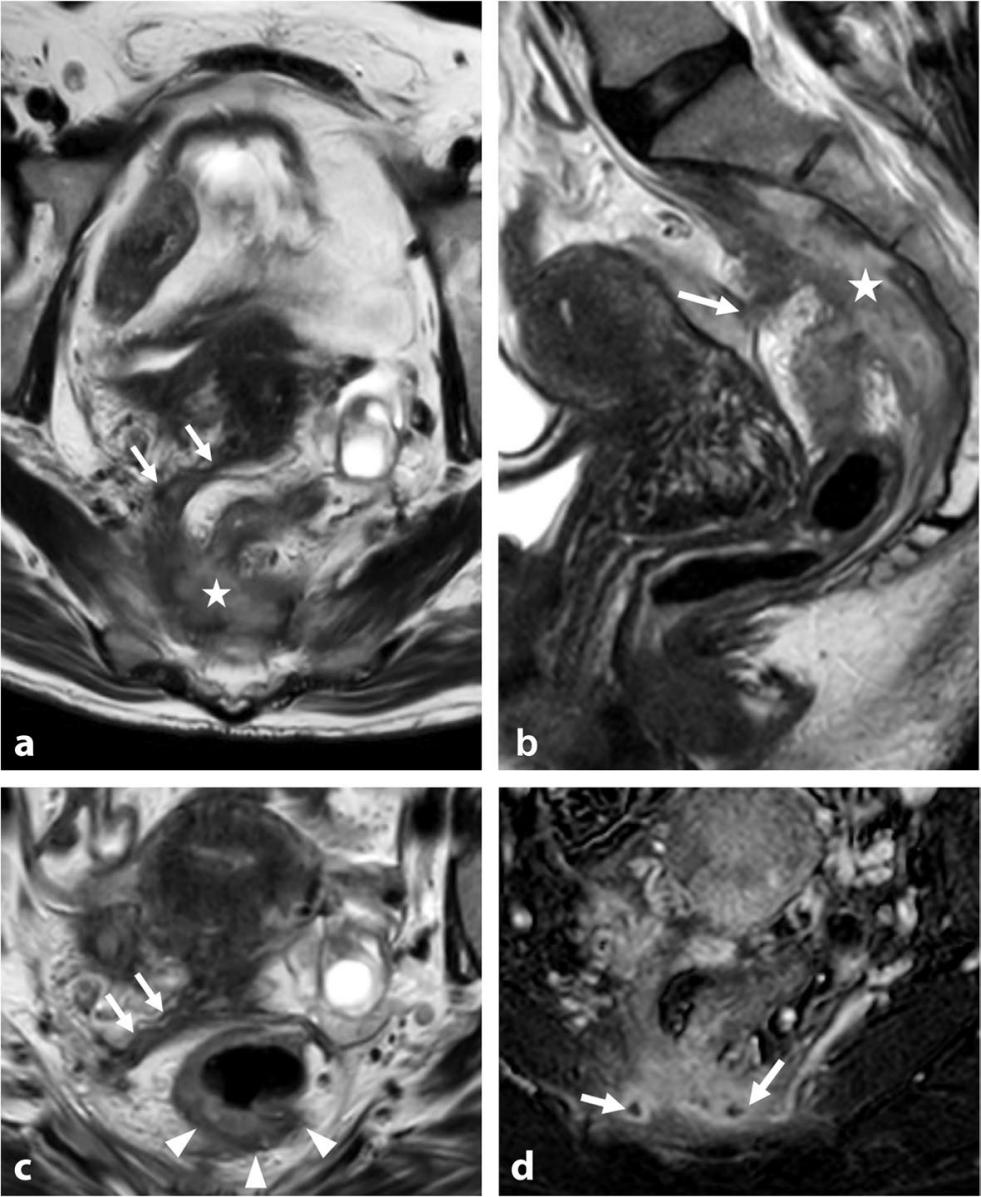
Actinomycosis in a 36-year-old woman with chronic pelvic pain, rectal disorder. and dyspareunia. **a** Axial and (**b**) sagittal T2-W MR images show right subperitoneal infiltration with intermediate signal intensity, centered on the right utero-sacral ligament (thin arrows) and the sacro-recto-genital septum up to the presacral space (star) from the first sacral vertebra to the sacrococcygeal junction. **c** Axial T2-W MR image shows perirectal soft-tissue infiltration (arrows) with intermediate T2-signal intensity semicircumferential thickening of the upper and posterior rectum (arrowheads). **d** Axial T1-W fat-suppressed contrast-enhanced subtracted MR image shows avid enhancement of the surrounding inflammatory infiltration with small abscesses in the presacral space (arrows). Past medical history of intrauterine device recent removal was found

Pelvic actinomycosis shows prominent fibrotic tissue and inflammatory stranding, resulting in intermediate to low signal intensity on T2-WI. The mass exhibits mild to marked enhancement, aiding in the differential diagnosis (Fig. 9). High signal intensity components on T1-WI due to hyperproteic content or free radicals are rare, with mild intensity seen within necrotic areas. Surgical intervention should be avoided, and a CT-guided needle biopsy is preferred for definitive microbiological diagnosis prior to initiating medical treatment.

#### Alveolar echinococcosis

**Fig. 10:**
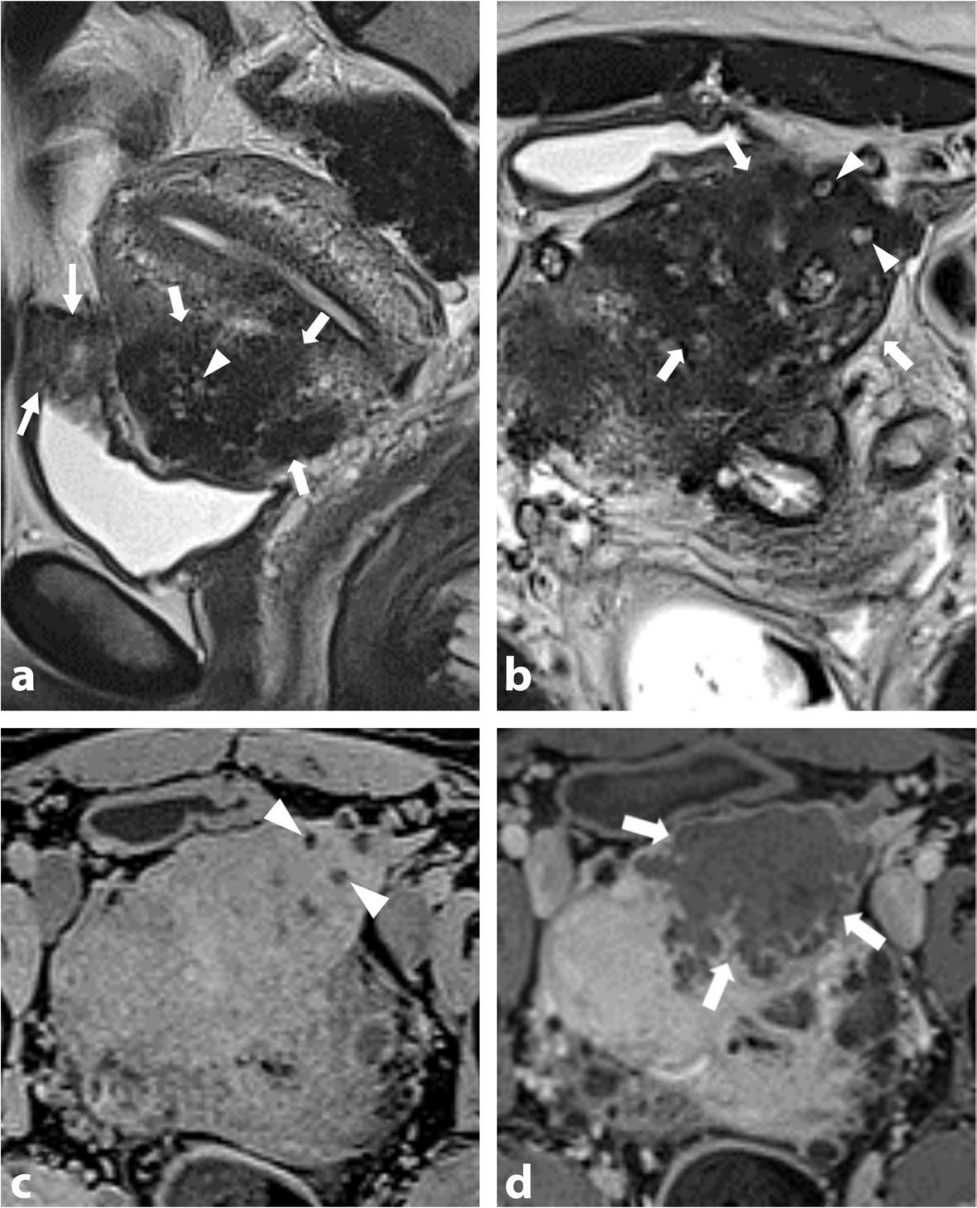
Pelvic alveolar echinococcosis in a 28-year-old woman with chronic pelvic pain, bladder disorder, and dyspareunia. **a** Sagittal and (**b**) axial T2-W MR images show an infiltrative T2-hypointense external myometrial infiltrative mass (thick arrows) with a few microcysts (arrowheads), and a contiguous infiltration of the bladder dome (thin arrows). **c** Axial T1-W fat-suppressed MR image shows several microcysts (arrowheads) without hemorrhagic foci in the extrinsic infiltrative uterine mass. **d** Axial T1-W fat-suppressed contrast-enhanced subtracted MR image shows central necrosis (arrows) and irregular margin with a peripheral enhancement. History of liver alveolar echinococcosis infection in childhood was then found

Human echinococcosis is a parasitic disease, or zoonosis, with endemic distribution in many parts of the world, including the Northern hemisphere. It is caused by *Echinococcus granulosus* that causes cystic echinococcosis and *Echinococcus multilocularis* that is the causative agent of alveolar echinococcosis [37]. The liver is the predominant initial site of parasitic development. Peritoneal and pelvic tissue involvement in alveolar echinococcosis is very rare, occurring through direct extension or peritoneal dissemination. The fibrotic reaction in the host can mimic DIE involvement of pelvic structures. MR key findings of alveolar echinococcosis include heterogeneous infiltrating multivesicular masses with irregular margins, along with T2-hypointense fibrotic components [38]. Small cystic and/or necrotic T2-hyperintense components may be seen, but hemorrhagic foci are missing on T1-W sequence. Necrosis may be seen in the center of the lesions as areas of low to intermediate signal intensity on T1-WI and heterogeneous signal intensity on T2-WI (Fig. 10). Calcifications may be seen in chronic pelvic fibrotic lesions on CT scan.

Alveolar echinococcosis can resemble profuse and severe DIE, but there are distinguishing features. While endometriosis causes distortion of the pelvic cavity with solid lesions and fibrous tissue reaction, echinococcosis presents as a multivesicular pattern with no substantial or faint long-lasting peripheral enhancement on contrast-enhanced images. Co-existence of multicystic masses in both the pelvic and hepatic regions is pathognomonic for alveolar echinococcosis. In contrast, endometriosis does not infiltrate liver parenchymal tissue in the same manner and typically originates along the hepatic capsule rather than forming circumscribed masses within the liver.

## Feces

**Fig. 11:**
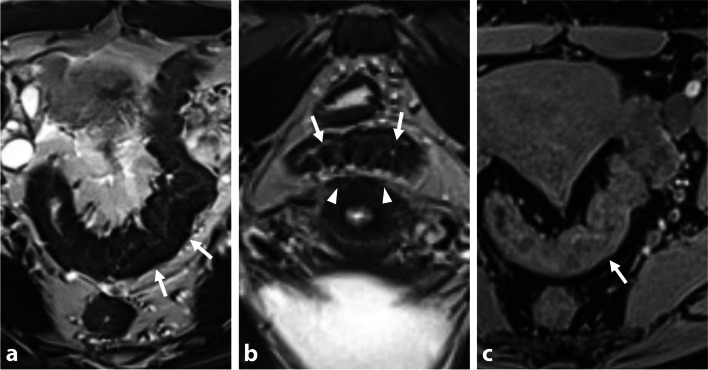
Normal sigmoid colon filled with feces in a 35-year-old woman with chronic pelvic pain. No medical history. **a** Axial and (**b**) coronal T2-W MR images show a T2-hypointense “pseudo”-thickening of the posterior sigmoid colon wall (arrows). Note the absence of retrocervical deep infiltrating endometriosis (arrowheads). **c** Axial T1-W fat-suppressed MR image shows the absence of hyperintense foci nor abnormality of the sigmoid colon wall (arrow), with a more or less fecal content. Laparoscopy showed a normal recto-sigmoid colon

Dehydrated solid feces can sometimes be misleading when evaluating bowel endometriosis, as they appear as low signal intensity on T2-WI. Multiplanar analysis using T2-W and 3D T1-W sequences helps for precise endoluminal location of feces, depicting a slightly heterogeneous signal on T1-WI, in contrast to extrinsic fibrotic bowel involvement in DIE (Fig. 11). Large folds or a wrinkled appearance of the rectum or sigmoid wall may occasionally be mistaken for endometriotic involvement, particularly in the sagittal plane. However, pseudo-thickening is typically present in only one plane with the same signal as the contiguous intestinal wall. Recent European recommendations [39] highly recommend bowel preparation and additional fasting prior to pelvic MRI in the evaluation of rectosigmoid endometriosis. The use of rectal opacification with sonographic gel and/or water is optional, with varying results reported for assessing the pouch of Douglas and rectosigmoid endometriosis according to different studies [40]. If there is uncertainty, a dedicated transvaginal ultrasound for endometriosis can be considered.

## Surgical material: vesicoureteral reflux and incontinence treatments

**Fig. 12:**
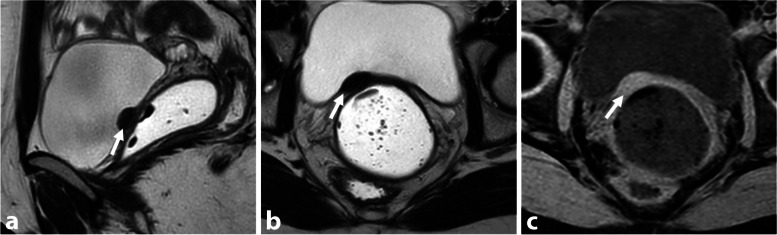
Unilateral right-sided vesicoureteral reflux surgical bulking agent (Macroplastique®—polydimethylsiloxane injection) in a 26-year-old woman. **a** Sagittal and (**b**) axial T2-W MR images show a T2-hypointense unilateral right-sided vesicoureteral reflux implant (arrows) at the ureterovesical junction. **c** Axial T1-W fat-suppressed MR image shows an ovoid geometrical shape in T1-isosignal intensity (arrow)

The endoscopic treatment of vesicoureteral reflux, primarily performed in childhood, can be encountered in adult patients undergoing evaluation for endometriosis [41]. The presence of injected bulking agents or synthetic graft material at the ureterovesical junction, or slightly behind in the pre-vesical terminal ureter, can potentially lead to a misdiagnosis of DIE involving the parametrium (Fig. 12). Implants, particularly collagen materials, can exhibit low signal intensity on T2-W sequences, resembling endometrioid implants with surrounding tissue granulomas [42]. These implants are typically challenging to visualize on T1-WI and fat-saturated T1-WI, often not visible or best depicted in isosignal. Imaging key features such as bilateral and symmetric pattern, geometric shape, in the absence of distortion or extrinsic infiltration helps in the correct diagnosis (Supplemental – Fig. 6). If there is uncertainty regarding the presence of surgical material, a pelvic CT scan can be useful in visualizing calcifications and hyperdense foreign materials. Additionally, with the same MRI appearance as the implants mentioned above, peri-urethral injections for the treatment of incontinence in adult women has increased in recent years and should not be confounded for endometriosis [43] (Supplemental –Fig. 7).

## Conclusion

In conclusion, the diagnosis of deep pelvic infiltrating endometriosis requires careful consideration of a wide range of differential diagnosis on MRI. It is important to be aware of both pathological and non-pathological conditions that can mimic endometriosis. Among these, injection of gadolinium may be useful to reach precise diagnosis, but must remain justified, as systematic injection is not recommended. While endometriosis is prevalent, it is crucial to appropriately communicate and consider alternative diagnosis. Incorrect diagnosis can result in unnecessary medical and surgical interventions that may have long-term consequences. It is vital to understand the strengths and limitations of MRI in diagnosing endometriosis to ensure accurate diagnosis and appropriate treatment decisions.

### Supplementary Information

**Additional file 1: Figure 1. **Bilateral thickening of the round ligaments in a 36-year-old woman. No medical history. **Figure 2.**  Uterine contraction in a 24-year-old woman, addressed for suspicion of endometriosis. **Figure 3.** Uterine retroversion surgery in a 38-year-old woman with anterior pelvic pain. **Figure 4.** Urinary bladder leiomyoma in a 35-year-old woman with chronic pelvic pain and bladder disorder. **Figure 5.** Vaginal leiomyoma in a 42-year-old woman with dyspareunia and vaginal palpable mass. **Figure 6.** Bilateral vesicoureteral reflux surgical implants in a 32-year-old woman. **Figure 7.** Urethral bulking agent (collagen) injection for the treatment of stress urinary incontinence in a 34-year-old woman.

## Data Availability

The data of cases in the manuscript are available from the corresponding author on reasonable request.

## Abbreviations

ADC: Apparent diffusion coefficient
DIE: Deep infiltrating endometriosis
DWI: Diffusion-weighted imaging
FS T1-WI: Fat-suppressed T1-weighted images
MRI: Magnetic resonance imaging
T1 T2-WI: T1 T2-weighted images
USL: Uterosacral ligament
