# Correction: Endometriosis MR mimickers: T2-hypointense lesions

**DOI:** 10.1186/s13244-024-01674-z

**Published:** 2024-03-25

**Authors:** Edouard Ruaux, Wendaline M. VanBuren, Stéphanie Nougaret, Marie Gavrel, Mathilde Charlot, Flavia Grangeon, Pierre-Adrien Bolze, Isabelle Thomassin-Naggara, Pascal Rousset

**Affiliations:** 1 Department of Radiology, Hospices Civils de Lyon, Lyon Sud University Hospital, Lyon 1 Claude Bernard University, EMR 3738 Pierre Bénite, France; 2 Department of Radiology, Mayo Clinic, Rochester, MN55905 USA; 3 Department of Radiology, Montpellier Cancer Institute, U1194, Montpellier University, 34295 Montpellier, France; 4 Department of Gynecology and Obstetrics, Hospices Civils de Lyon, Lyon Sud University Hospital, Lyon 1 Claude Bernard University, EMR 3738, 69495 Pierre Bénite, France; 5 Department of Radiology, Service Imageries Radiologiques Et Interventionnelles Spécialisées, Hôpital Tenon, Assistance Publique Hôpitaux de Paris, Sorbonne Université, 75020 Paris, France

**Correction: Insights into Imaging 15, 20 (2024)**

**https://doi.org/10.1186/s13244-023-01588-2**

The original article [1] contained a minor typo in the graphical abstract which has since been amended.
