# Supplementary material for: Endometriosis MR mimickers: T2-hypointense lesions
Source: Insights Imaging. 2024 Jan 25;15:20. doi: 10.1186/s13244-023-01588-2 (PMC10808434; doi:10.1186/s13244-023-01588-2)
Supplement: Supplementary file 1 — Additional file 1: Figure 1. Bilateral thickening of the round ligaments in a 36-year-old woman. No medical history. Figure 2. Uterine contraction in a 24-year-old woman, addressed for suspicion of endometriosis. Figure 3. Uterine retroversion surgery in a 38-year-old woman with anterior pelvic pain. Figure 4. Urinary bladder leiomyoma in a 35-year-old woman with chronic pelvic pain and bladder disorder. Figure 5. Vaginal leiomyoma in a 42-year-old woman with dyspareunia and vaginal palpable mass. Figure 6. Bilateral vesicoureteral reflux surgical implants in a 32-year-old woman. Figure 7. Urethral bulking agent (collagen) injection for the treatment of stress urinary incontinence in a 34-year-old woman. [file 13244_2023_1588_MOESM1_ESM.docx]

**Endometriosis Mimickers: T2-Hypointense Lesions**

**ELECTRONIC SUPPLEMENTARY MATERIAL**

**
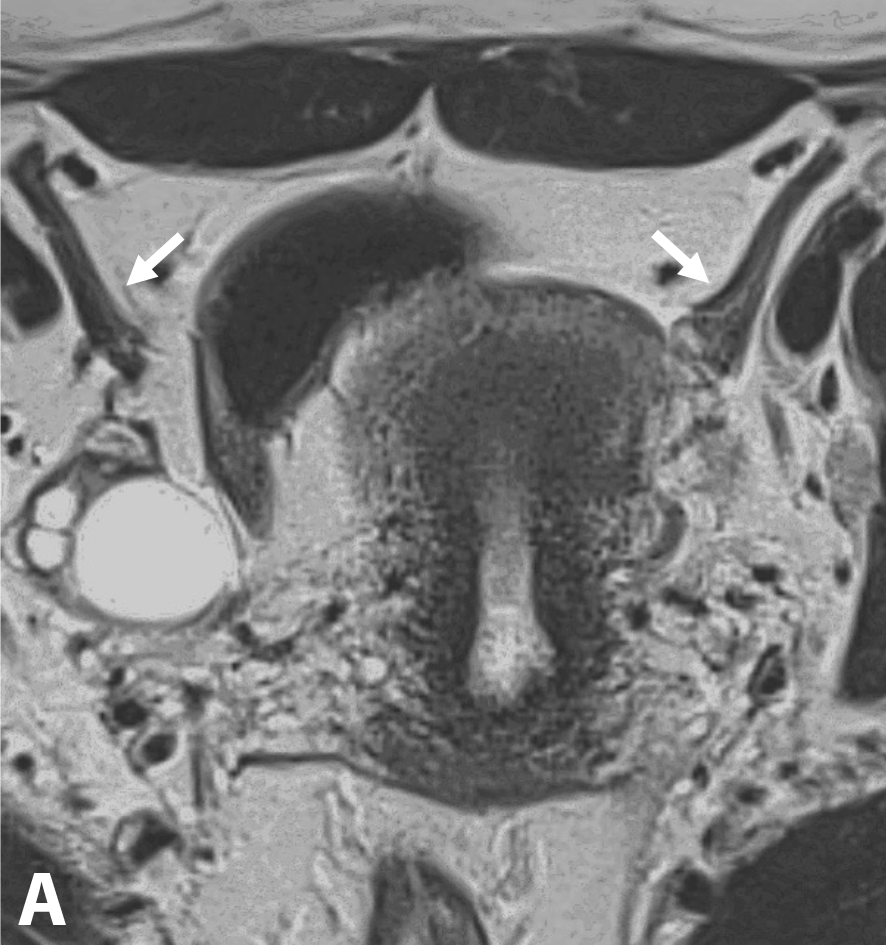

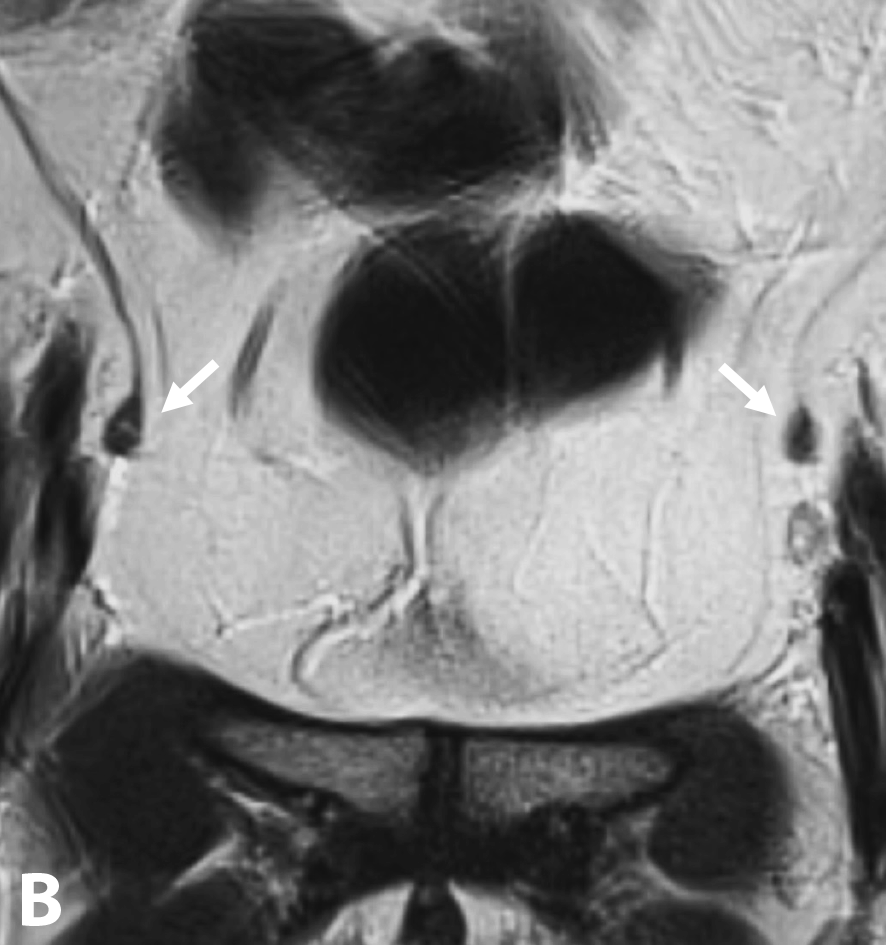

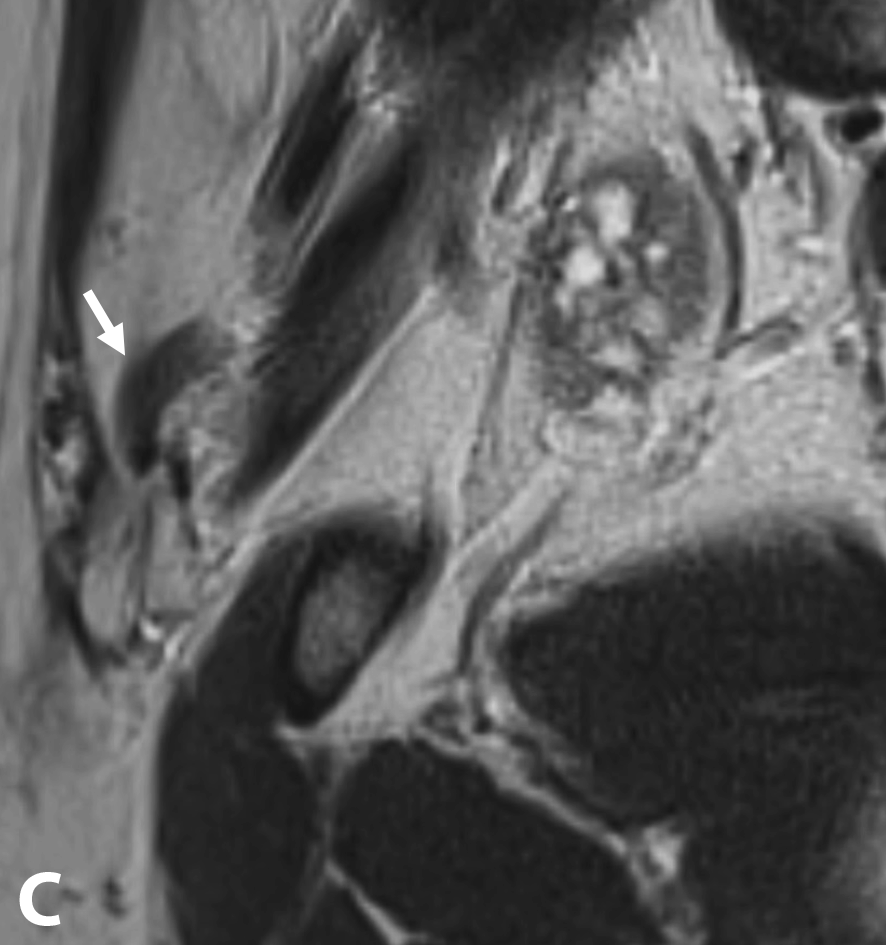
**

**Figure 1** - Bilateral thickening of the round ligaments in a 36-year-old woman. No medical history.

(a) Axial, (b) coronal and (c) sagittal T2-W MR images show bilateral round ligaments (arrows) with linear and regular thickening (< 1 cm).

No endometriosis was found at surgery.


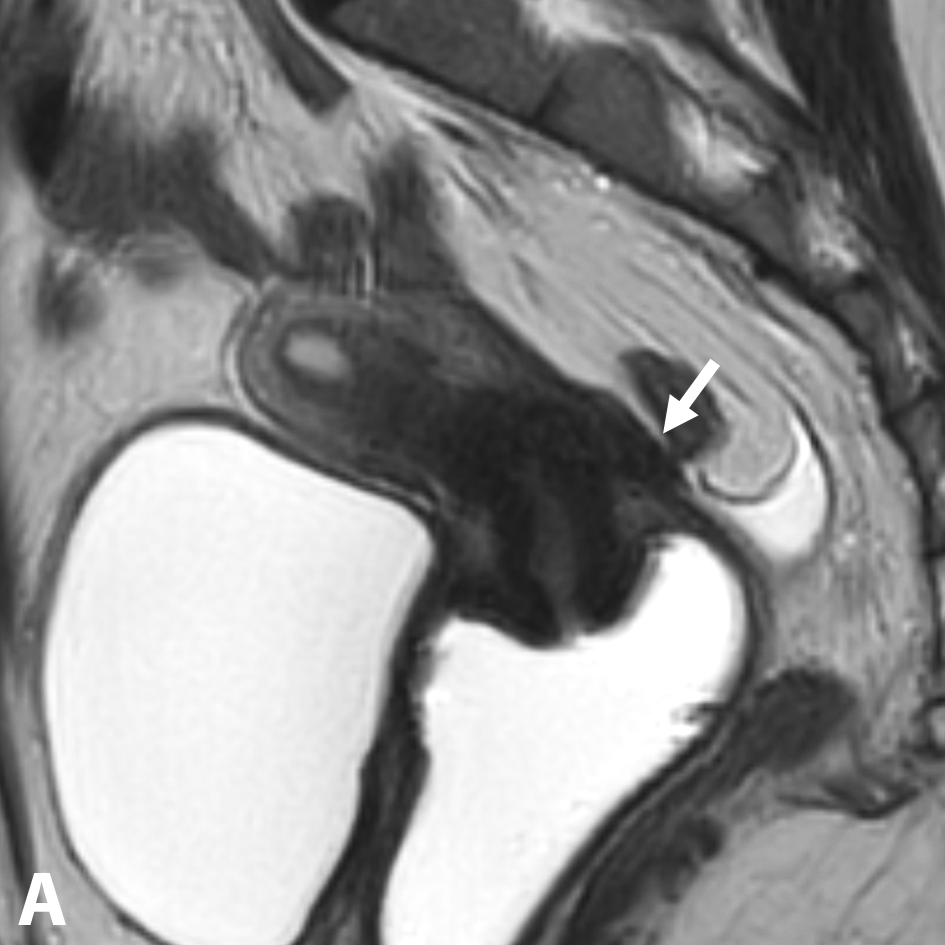

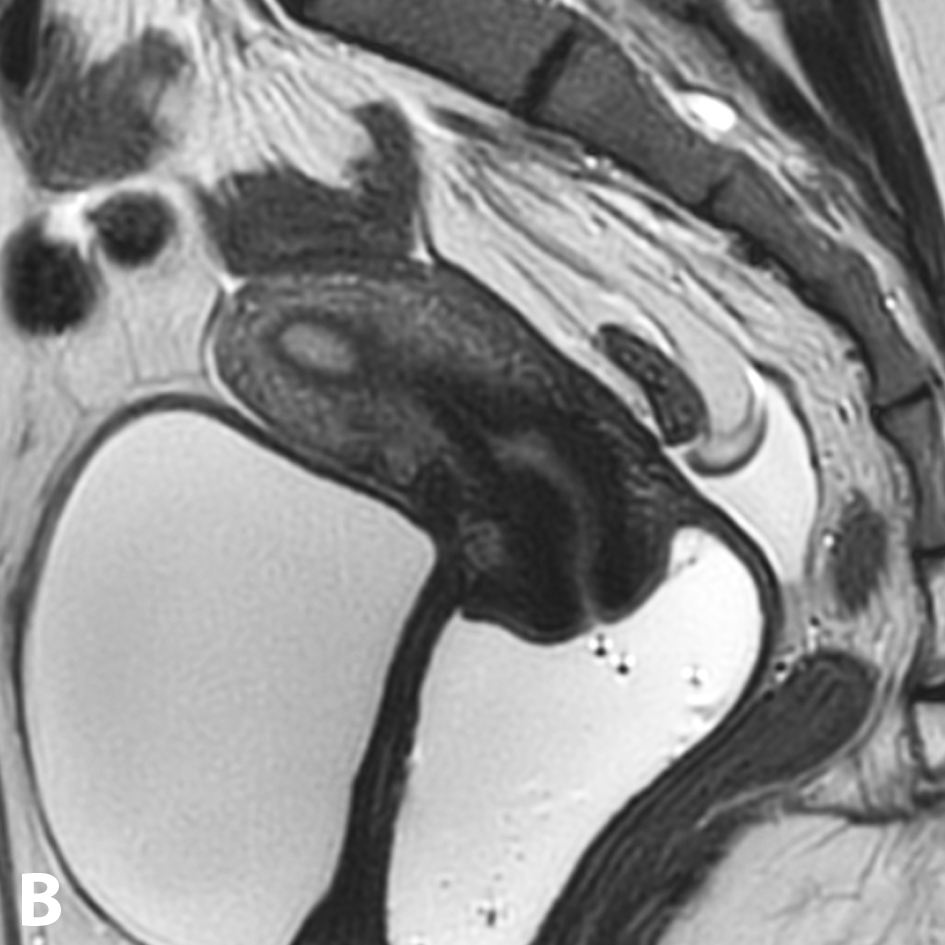


**Figure 2** - Uterine contraction in a 24-year-old woman, addressed for suspicion of endometriosis.

(a) Sagittal T2-W MR image shows a T2-hypointense focal thickening (arrow) in the outer myometrium, which appears opposite the torus and bears similarities to deep endometriosis.

(b) Note the absence of abnormality of the myometrium and its complete resolution on additional sagittal T2-W MR image repeated at the end of the exam.


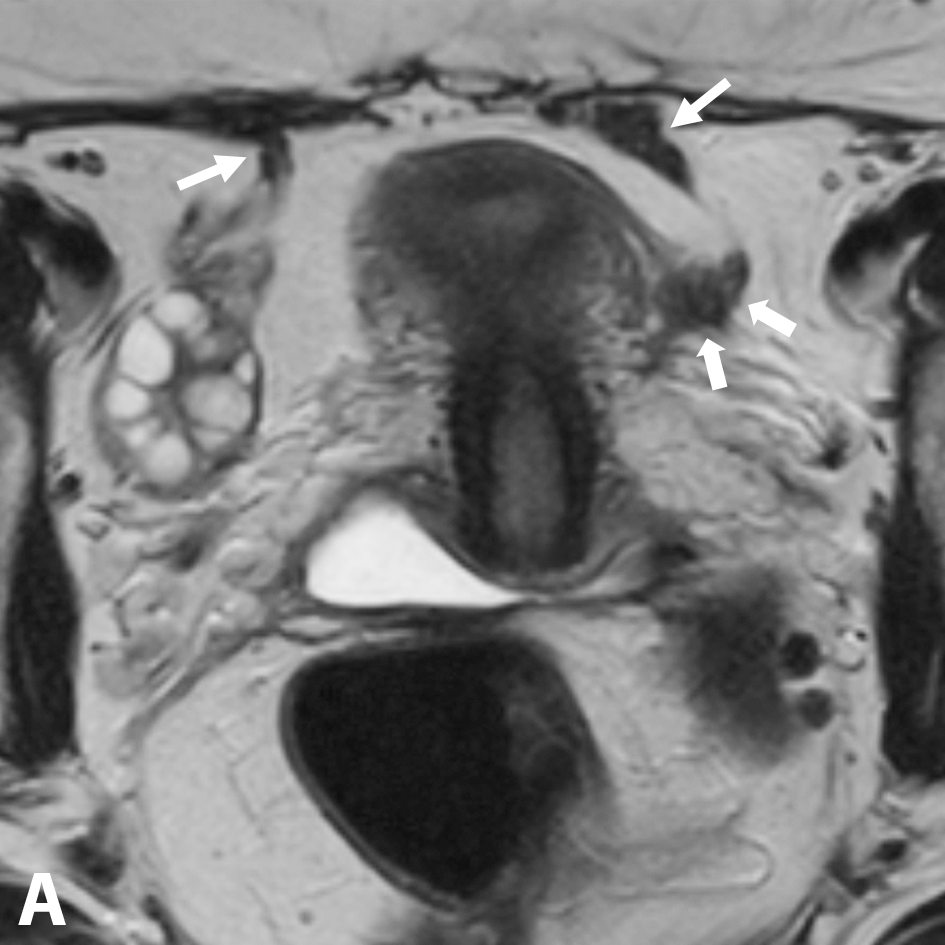

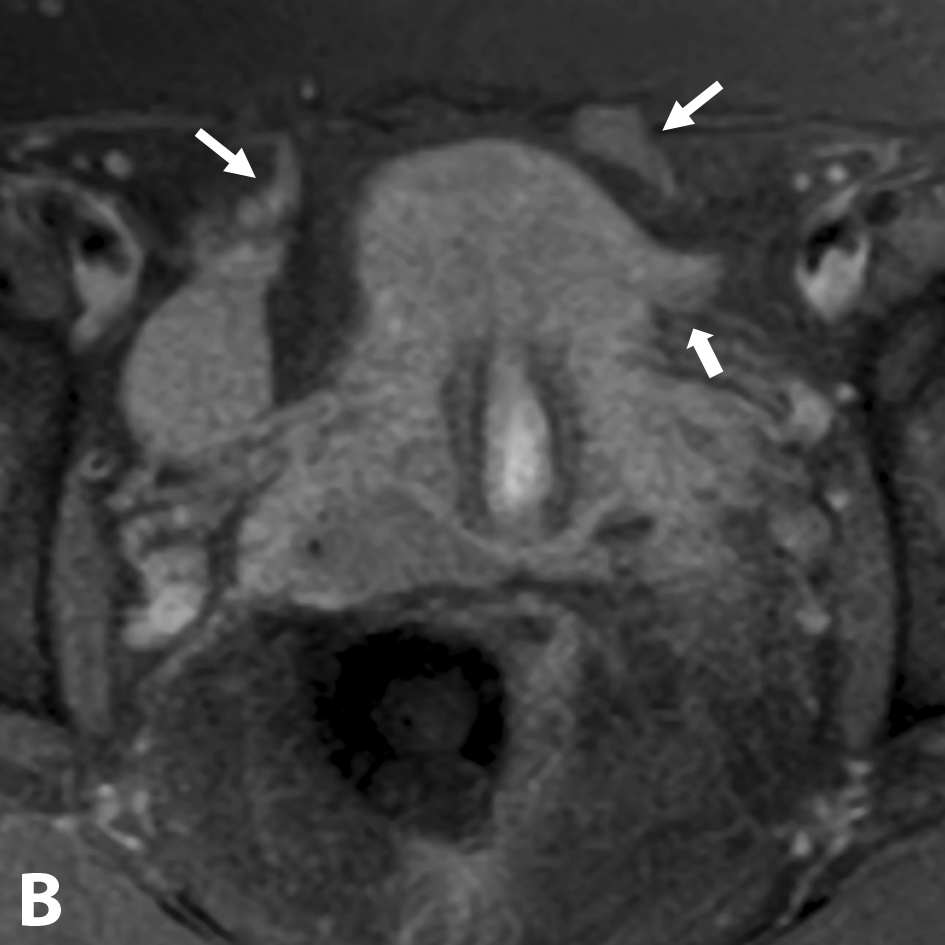
**Figure 3** - Uterine retroversion surgery in a 38-year-old woman with anterior pelvic pain.

(a) Axial T2-W MR image shows shortened round ligaments with a pseudonodular thickening of the left round ligament proximal section (thick arrows), up to their medial insertion on the pelvic wall (thin arrows).

(b) Axial fat-suppressed T1-W MR image reveals the absence of intralesional hemorrhagic foci within the left proximal round ligament (thick arrow) and both round ligaments medial insertion (thin arrows).


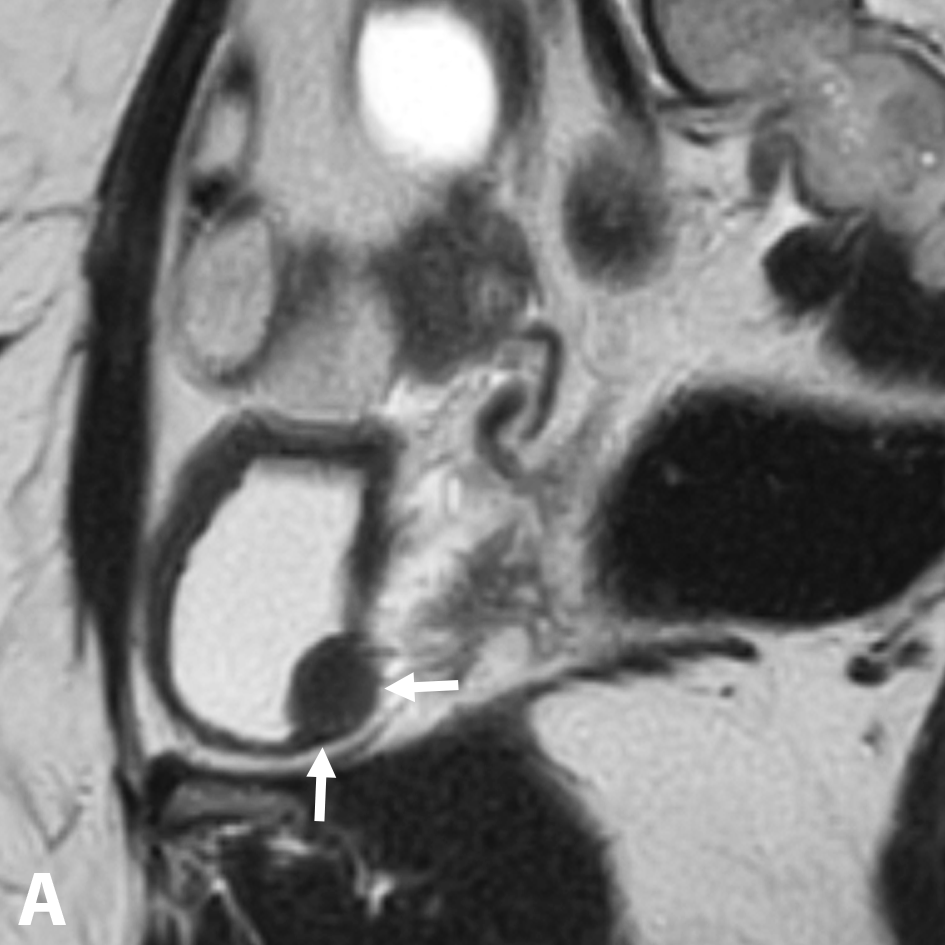

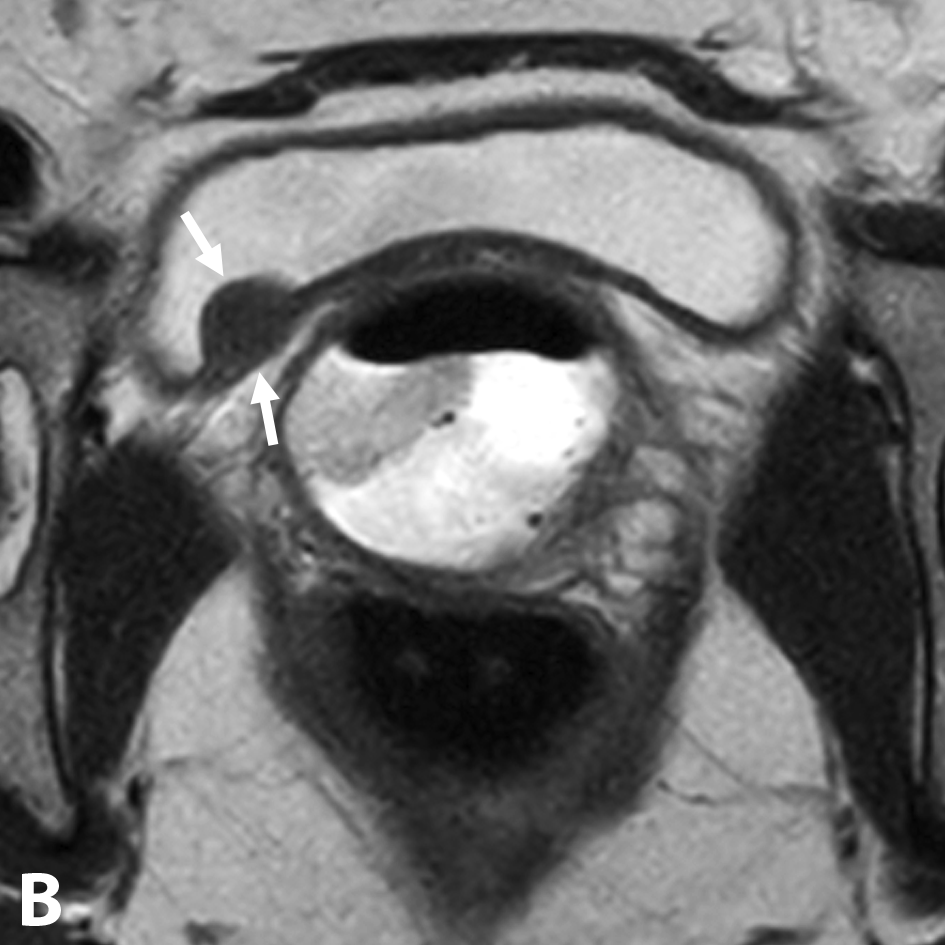


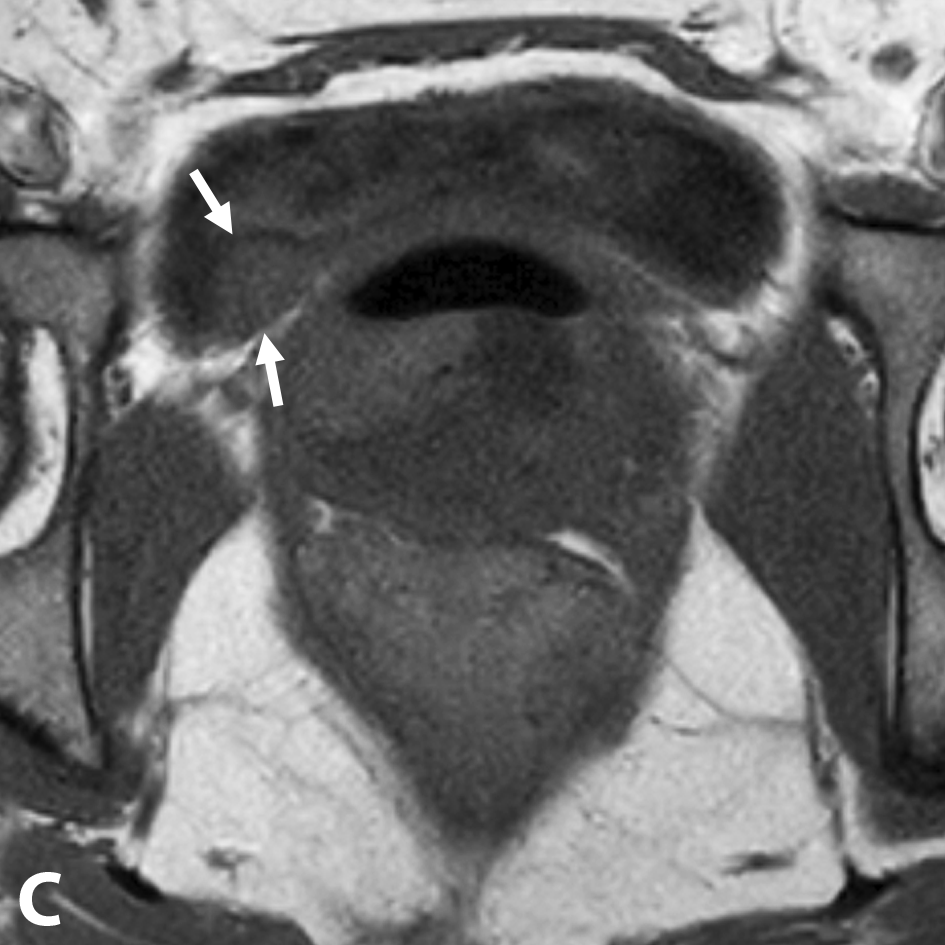

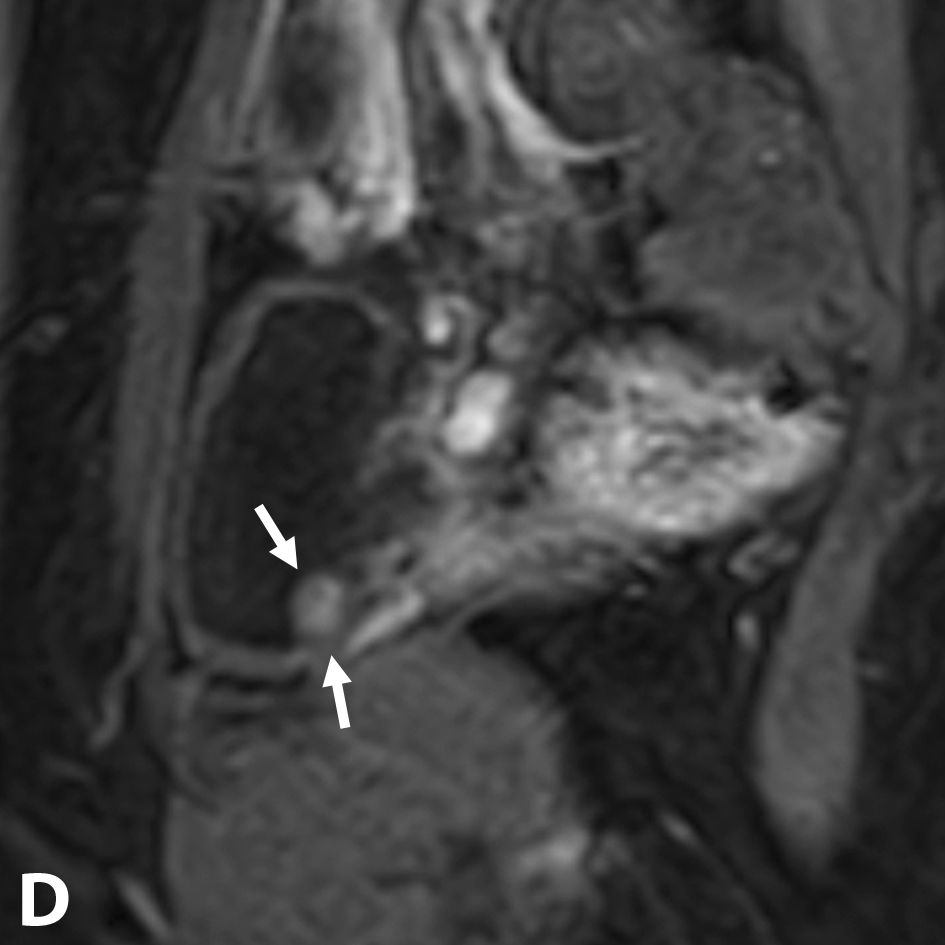


**Figure 4** - Urinary bladder leiomyoma in a 35-year-old woman with chronic pelvic pain and bladder disorder.

(a) Sagittal and (b) axial T2-W MR images show a well-defined rounded T2-hypointense bladder mass (arrows), located on the right side of the bladder base close to the ureterovesical junction.

(c) Axial T1-W MR image shows a parietal mass exhibiting T1-intermediate signal intensity (arrows) without hemorrhagic foci.

(d) Sagittal T1-W fat-suppressed contrast-enhanced MR image shows enhancement of the mass (arrows).

Diagnosis confirmed by cystoscopy biopsy.


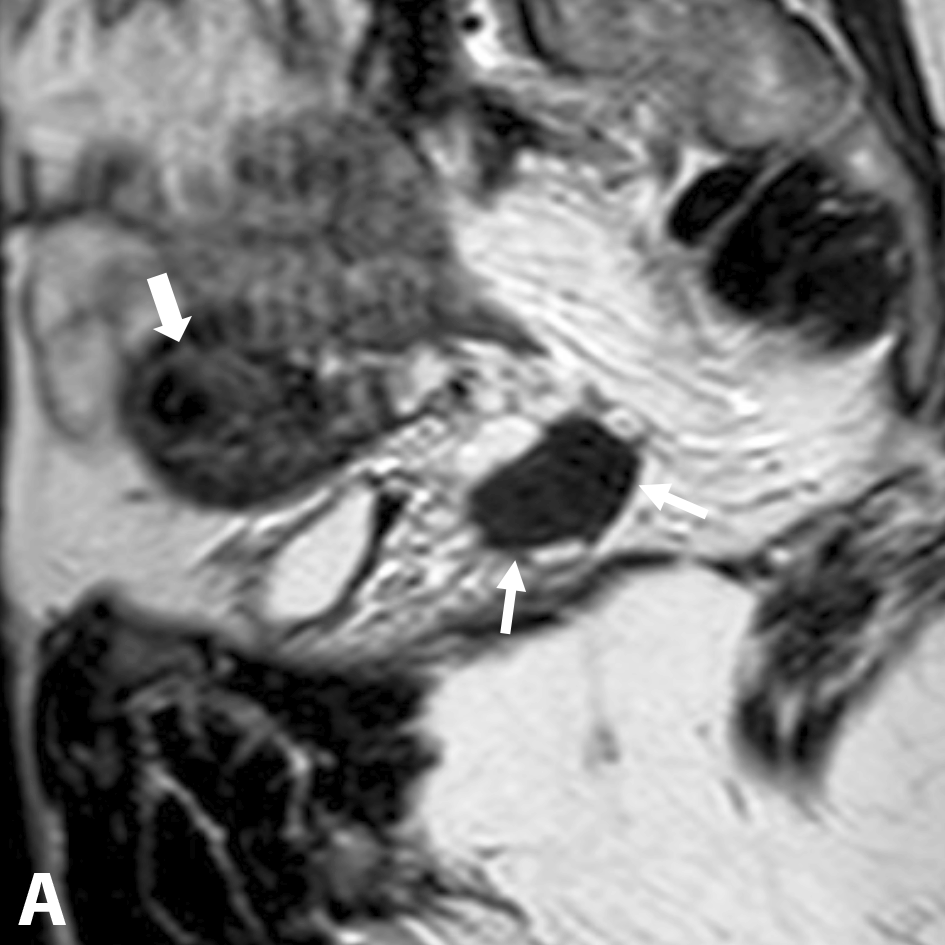

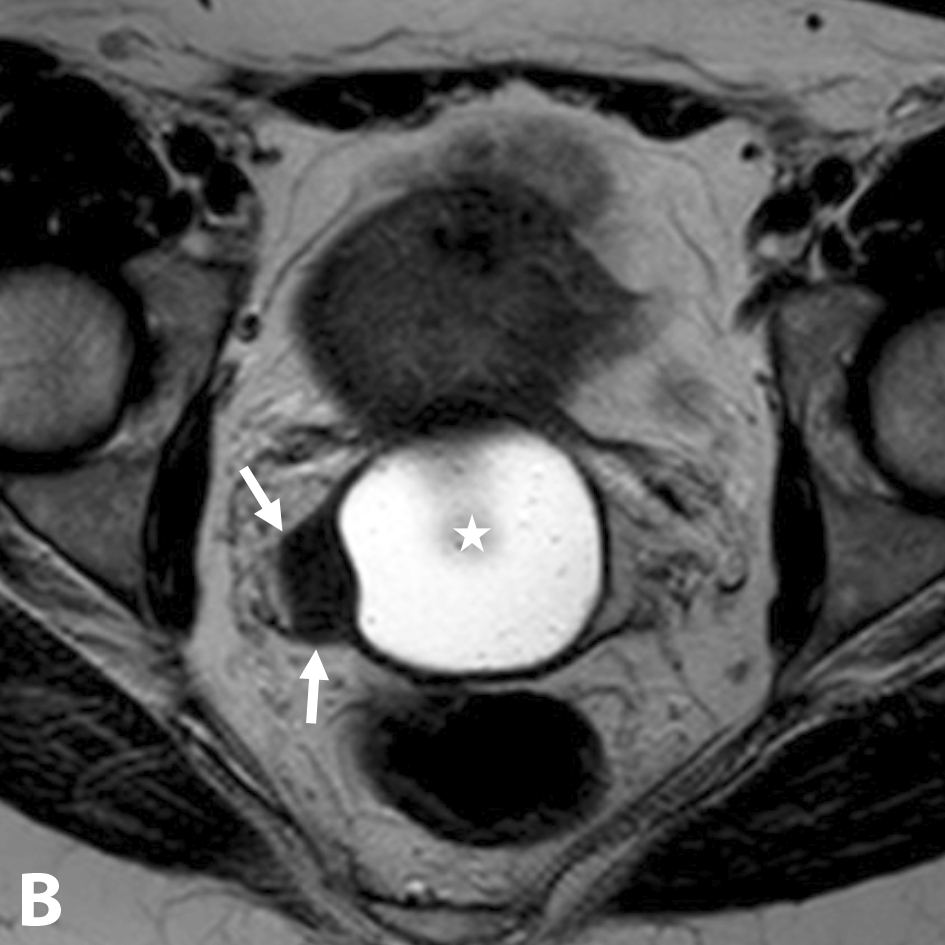


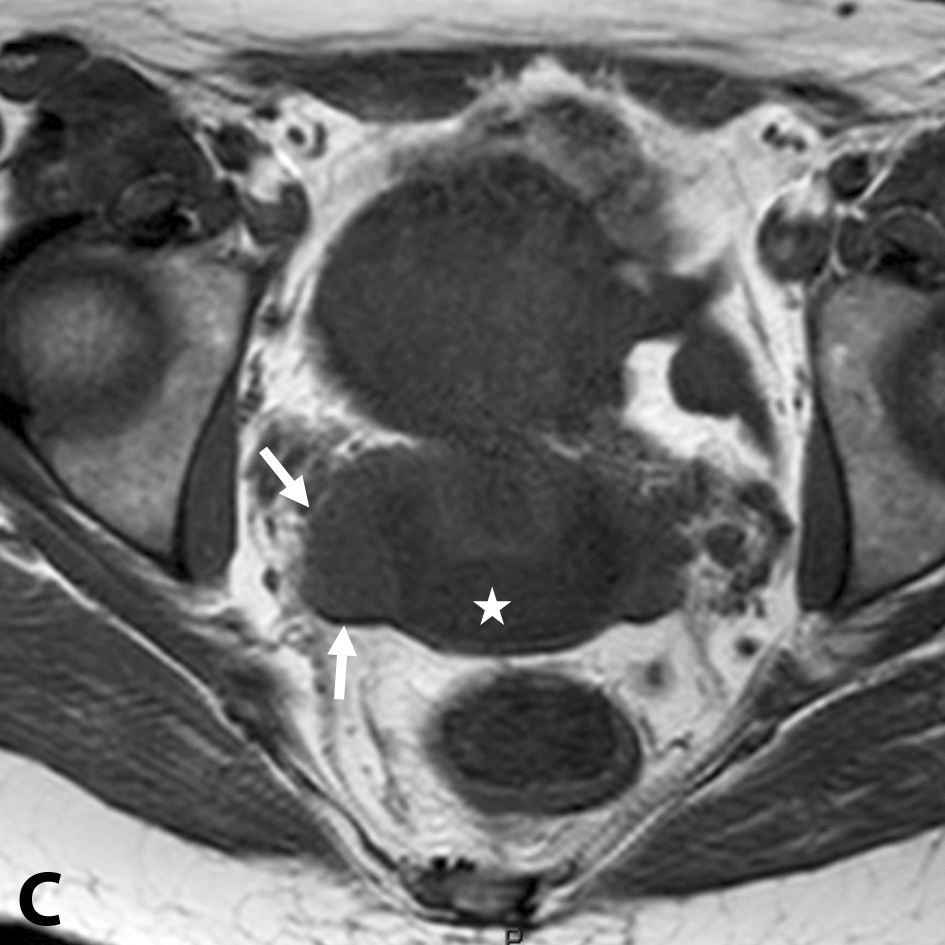

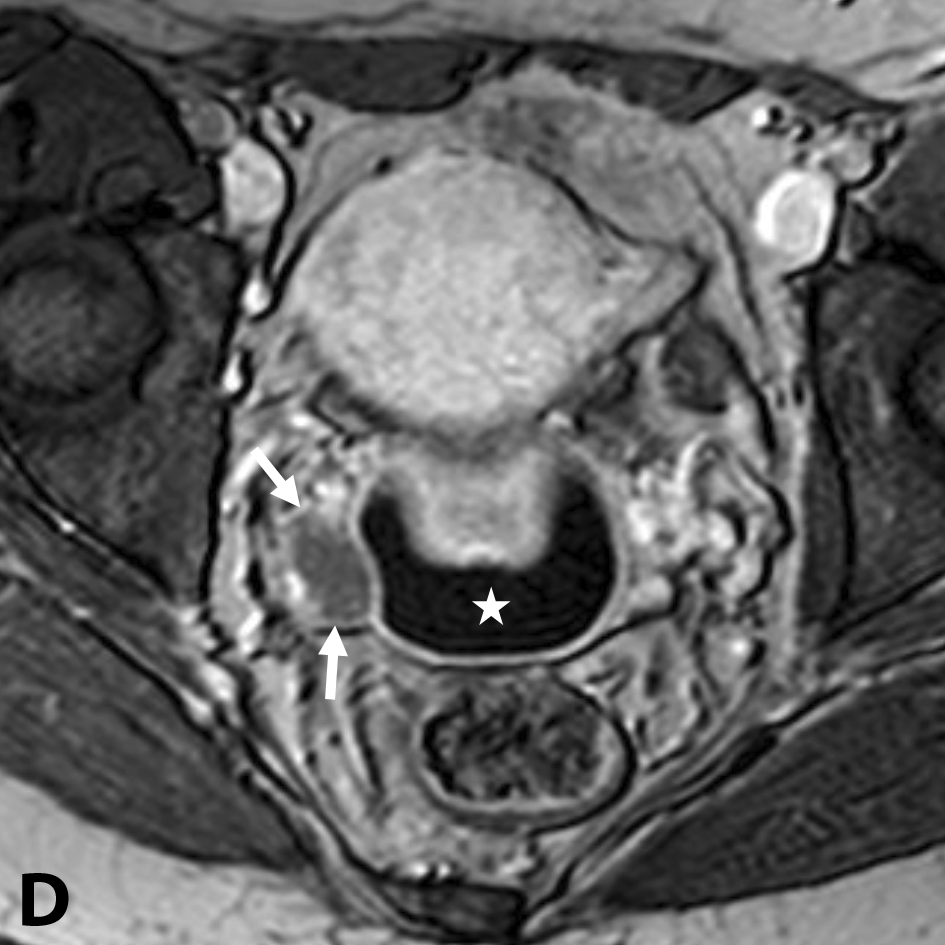


**Figure 5** - Vaginal leiomyoma in a 42-year-old woman with dyspareunia and vaginal palpable mass.

(a) Sagittal and (b) axial T2-W MR images show a well-defined vaginal rounded T2-hypointense mass (thin arrows), located on the right side of the posterior fornix. Note the uterus on the medial side of the mass (thick arrow).

(c) Axial T1-W MR image (including vaginal gel opacification (star)) shows a vaginal mass exhibiting T1-intermediate signal intensity (arrows) without hemorrhagic foci.

(d) Axial T1-W contrast-enhanced MR image shows noticeable but slight enhancement of the mass (arrows).

Surgical specimen was verified through examination in pathology.


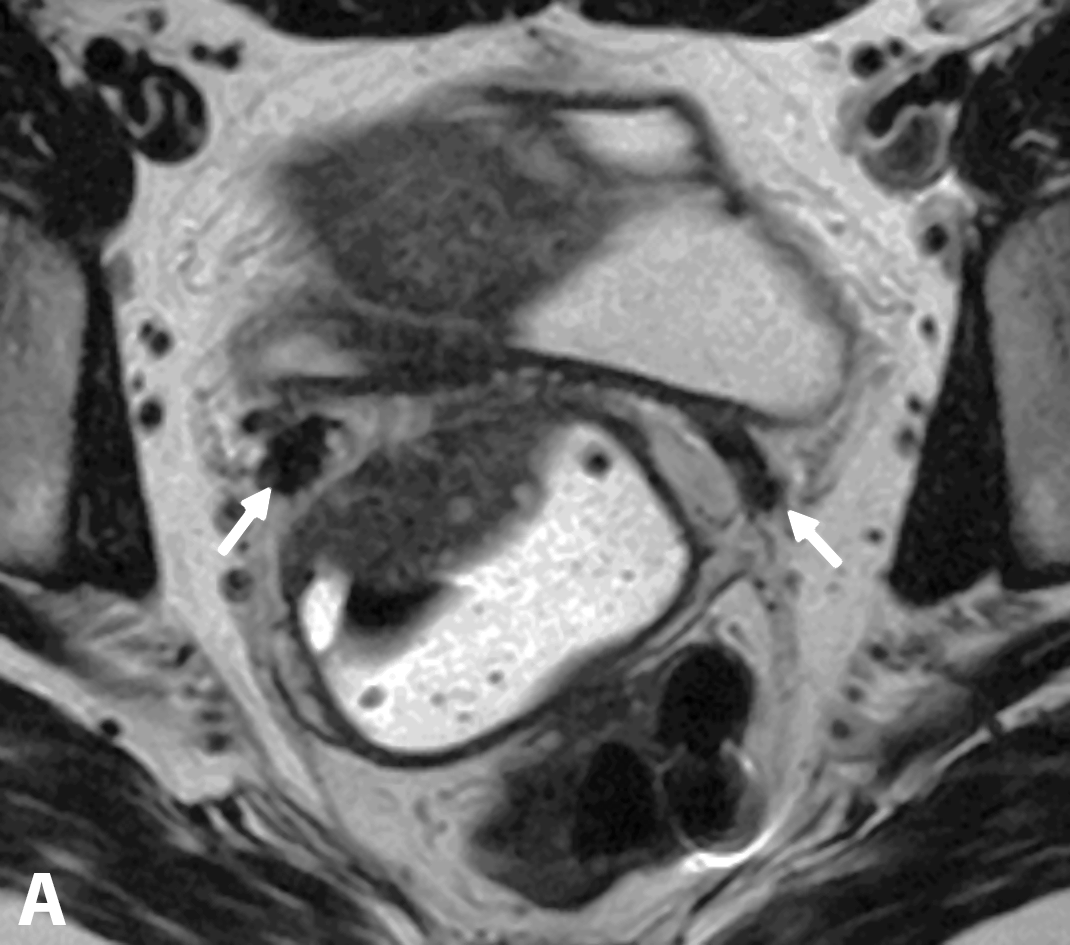


**Figure 6** - Bilateral vesicoureteral reflux surgical implants in a 32-year-old woman.

Axial T2-W MR image shows bilateral and symmetric T2-hypointense implants (arrows) behind the ureterovesical junction. Note the rectangular geometrical shape of the left implant and the absence of extrinsic infiltration or any distortion around.


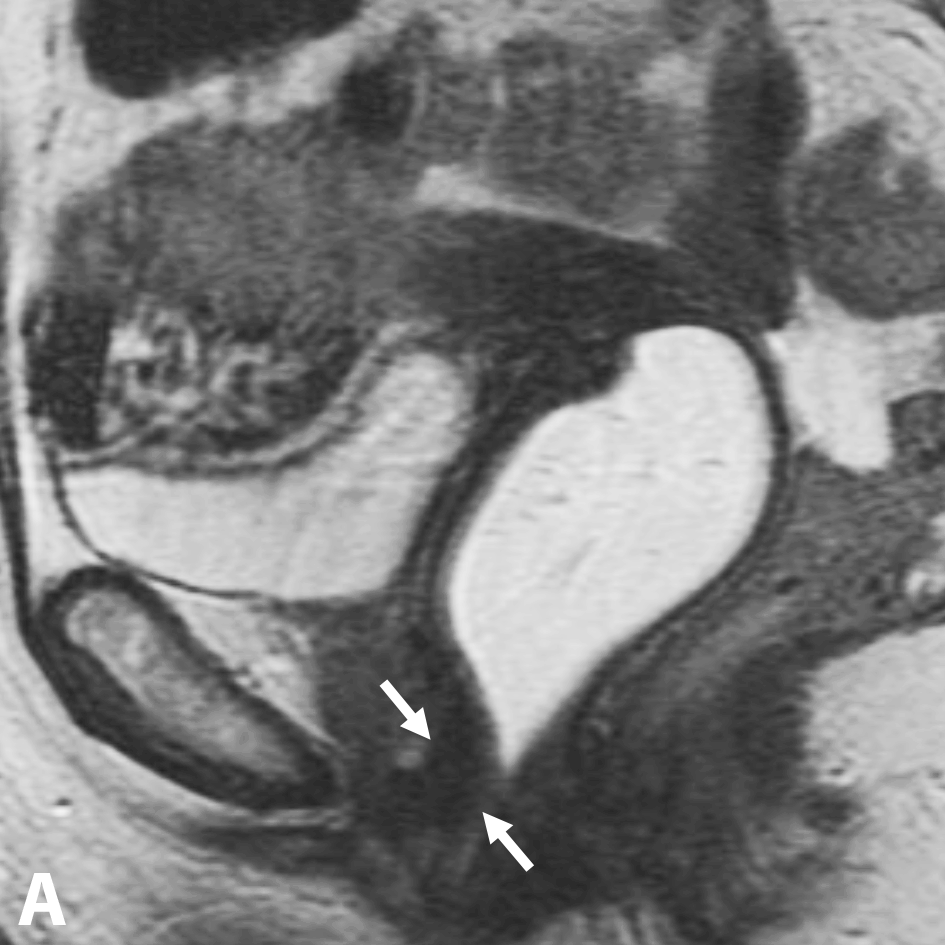

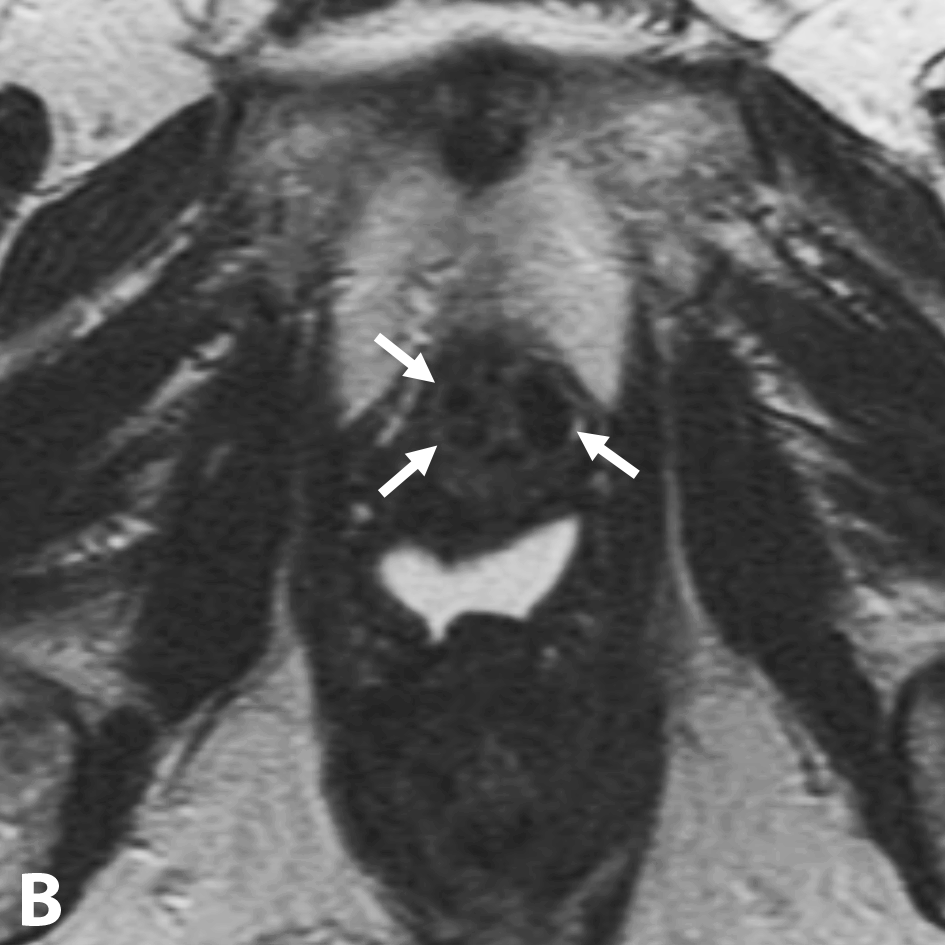


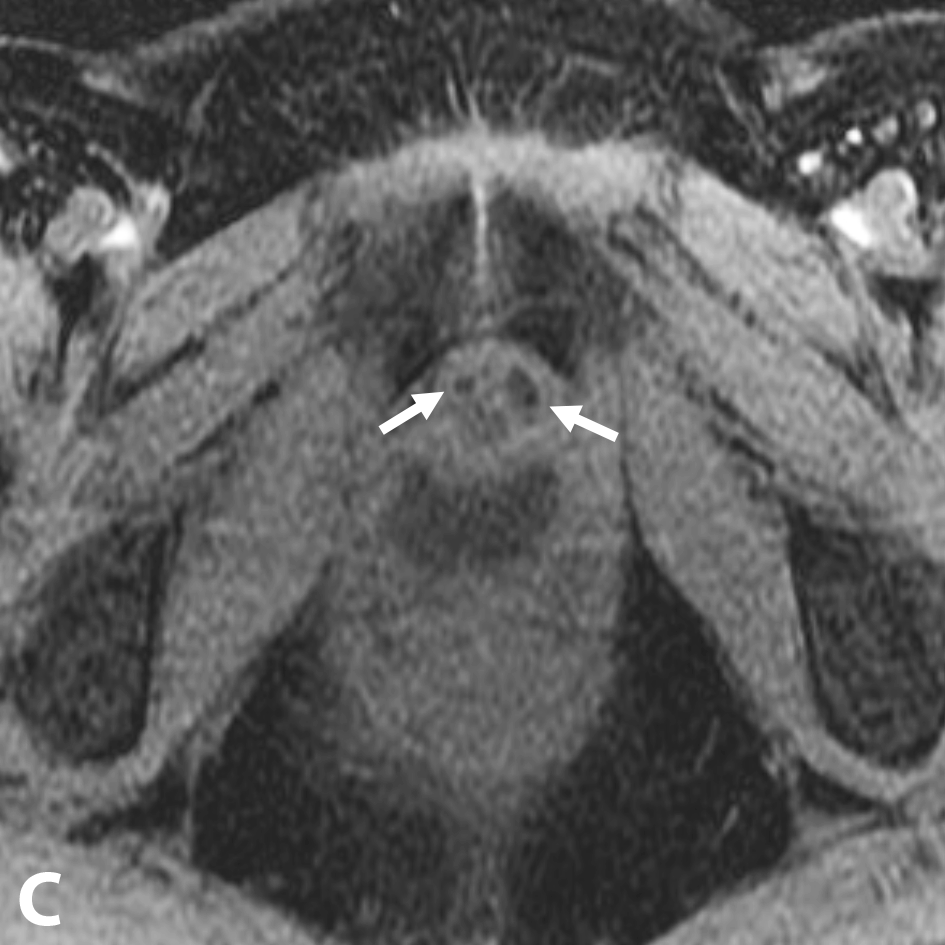


**Figure 7** - Urethral bulking agent (collagen) injection for the treatment of stress urinary incontinence in a 34-year-old woman.

(a) Sagittal and (b) axial T2-W MR images show a T2-hypointense collagen with a geometrical shape at different injection sites (arrows) within the urethral wall.

(c) Axial T1-W fat-suppressed MR image shows rounded T1-hypointense deposits (arrows).
